# Associating lncRNAs with small molecules via bilevel optimization reveals cancer-related lncRNAs

**DOI:** 10.1371/journal.pcbi.1007540

**Published:** 2019-12-26

**Authors:** Yongcui Wang, Shilong Chen, Luonan Chen, Yong Wang

**Affiliations:** 1 Key Laboratory of Adaptation and Evolution of Plateau Biota, Northwest Institute of Plateau Biology, Chinese Academy of Sciences, Xining, China; 2 Qinghai Provincial Key Laboratory of Crop Molecular Breeding, Northwest Institute of Plateau Biology, Chinese Academy of Sciences, Xining, China; 3 Key Laboratory of Systems Biology, Innovation Center for Cell Signaling Network, Institute of Biochemistry and Cell Biology, Shanghai Institutes for Biological Sciences, Chinese Academy of Sciences, Shanghai, China; 4 CEMS, NCMIS, MDIS, Academy of Mathematics and Systems Science, Chinese Academy of Sciences, Beijing, China; 5 Center for Excellence in Animal Evolution and Genetics, Chinese Academy of Sciences, Kunming, China; University of Ottawa, CANADA

## Abstract

Long noncoding RNA (lncRNA) transcripts have emerging impacts in cancer studies, which suggests their potential as novel therapeutic agents. However, the molecular mechanism behind their treatment effects is still unclear. Here, we designed a computational model to **A**ssociate **L**ncRNAs with **A**nti-**C**ancer **D**rugs (ALACD) based on a bilevel optimization model, which optimized the gene signature overlap in the upper level and imputed the missing lncRNA-gene association in the lower level. ALACD predicts genes coexpressed with lncRNAs mean while matching drug’s gene signatures. This model allows us to borrow the target gene information of small molecules to understand the mechanisms of action of lncRNAs and their roles in cancer. The ALACD model was systematically applied to the 10 cancer types in The Cancer Genome Atlas (TCGA) that had matched lncRNA and mRNA expression data. Cancer type-specific lncRNAs and associated drugs were identified. These lncRNAs show significantly different expression levels in cancer patients. Follow-up functional and molecular pathway analysis suggest the gene signatures bridging drugs and lncRNAs are closely related to cancer development. Importantly, patient survival information and evidence from the literature suggest that the lncRNAs and drug-lncRNA associations identified by the ALACD model can provide an alternative choice for cancer targeting treatment and potential cancer pognostic biomarkers. The ALACD model is freely available at https://github.com/wangyc82/ALACD-v1.

## Author summary

LncRNAs are RNA transcripts that are longer than 200 bp and do not encode proteins. Recent experimental studies have indicated the crucial role of lncRNAs in cancer. We proposed a computational model, ALACD, to understand a lncRNA’s molecular mechanism by associating it with a drug through the drug’s target genes. ALACD reveals lncRNAs, the associated anti-cancer drug, and the induced gene signatures that are involved in the regulation of cancer. Furthermore, these cancer-related lncRNAs are differentially expressed in cancer patients and closely associated with patient survival.

## Introduction

Human cancer is one of the leading causes of morbidity and mortality worldwide, and it involves multiple genetic, epigenetic, and transcriptional changes [1, 2]. Ongoing large-scale projects by some cancer genome consortiums, such as The Cancer Genome Atlas (TCGA), are using high-throughput molecular profiling strategies to characterize these changes. They first focus on decoding changes in protein-coding genes to interpret cancer genomics, and then shift their focus to the noncoding region due to the fundamental role of noncoding RNA in the regulation of a wide range of processes [3, 4], including cancer [5, 6]. Fox example, *HOX* transcript antisense RNA (*HOTAIR*) was highly expressed in breast cancer samples [7], Metastasis-Associated Lung Adenocarcinoma Transcript 1 (*MALAT1*) related with metastasis and survival in early-stage non-small cell lung cancer (NSCLC) [8], and Colon Cancer-Associated Transcript 2 (*CCAT2*) overexpressed in microsatellite-stable colorectal cancer [9]. Those findings indicate that lncRNAs are involved in the regulation of cancer development, and targeting them might provide a novel therapeutic strategy.

The modulatory mechanism of lncRNAs has advantages that support their potential as therapeutic targets. One of these advantages is that lncRNA expression is highly tissue- or cell- specific [10], which provides a great opportunity to develop therapeutics for targeting specific tissues. In addition, one of the main functions of lncRNAs is chromatin modification, thus targeting the interaction of lncRNAs with epigenetic factors, such as *PRC2*, could provide an efficient treatment approach. Moreover, many lncRNAs are located in the nucleus, and act as cis-regulatory elements for neighbouring genes [11], hence, gene locus-specific regulation can be achieved by lncRNA manipulation [12].

In addition, several strategies for lncRNA modulation have been developed. One of them is the application of specifically designed small interfering RNAs (siRNAs) to inhibit the function of lncRNAs, and the success of this strategy has been demonstrated [12]. Another approach is the oligonucleotide-based targeting of lncRNAs. Compared to siRNAs, oligonucleotides have higher specificity and fewer off-target effects due to the direct targeting of lncRNAs [13]. Collectively, these findings encourage the study of lncRNAs in the treatment of cancer patients. Various lncRNAs that target therapeutic agents are being investigated, and several companies have attempted to develop lncRNA-targeting therapeutics for the treatment of human diseases, including cancers [14, 15]. Moreover, some computational works have also attempted to address this topic to further understand the molecular mechanism of lncRNA in cancer.

The current computational works for this topic can be divided into three types. One type attempts to identify cancer-related lncRNAs, which exhibit significantly different expression levels in cancer patients, by comparing gene expression in tumors and normal tissues of cancer patients. The aim of this type of works is to find differentially expressed lncRNAs that are link to cancer, such as TANRIC [16]. The second aim is to attempt to predict the associations between small molecules and lncRNAs through linking drug response with RNA expression ([17–20]). The lncRNA signatures are generated through identifying the lncRNAs that display significantly different expression levels before/after small molecule treatment. This type of work actually reveals lncRNAs that are affected by drug treatment. The third aim is to derive the information from some mediator (such as miRNA), and apply the associations between lncRNAs and that mediator to transfer disease information from that mediator to lncRNA ([21–24]).

Although both wet-bench and computational experiments were utilized to reveal the associations between lncRNA and cancer, there is still a large gap between existing knowledge and clear picture of the mechanism of action of lncRNAs in cancer. However, chemical therapies have been well studied, and recent high-throughput drug screening technologies have generated genomic data, and pharmacological profiling of hundreds of compounds across thousands of cancer cells [25–29]. In addition, some curated databases have deposited multiple-platform data sources, which describe drug functions in living cells. They include drug chemical structure, target protein, side-effects, therapeutic annotations (ATC-code), *etc*. [30, 31]. Jointly, these valuable data hint the mechanism of action of drugs in cancer [32, 33]. Thus, associating lncRNAs with drugs may provide a deeper understanding of the mechanisms of action of lncRNAs and their roles in cancer.

Here, we developed a systematically computational approach for **A**ssociating **L**ncRNAs with **A**nti-**C**ancer **D**rugs (ALACD) via a bilevel optimization. The model first identified drug-induced gene-expression signatures through expression analysis of the Connectivity Map (CMap) data [26]. Then, we calculated the expression correlations between lncRNAs and mRNAs in patients from The Cancer Genome Atlas (TCGA), and further expanded those correlations through a supervised learning algorithm, the Support Vector Machines (SVM), which is motivated by statistical learning theory [34, 35]. Finally, through bilevel optimization, lncRNAs, the associated anti-cancer drugs, and the induced gene signatures involved in the regulation of cancer, were uncovered.

## Materials and methods

### Anti-cancer drugs

The Connectivity Map (CMap, build 02) data [26], which contains 6,100 gene expression profiles of 4 cell lines treated with 1,309 distinct small molecules with diverse doses, was applied to detect drug-induced gene signatures. The histogram of treatment instances with respect to the drugs in CMap is presented in S1 Fig. To achieve differentially expressed genes (DEGs) with much more significance, 29 drugs with more than ten treatment instances were selected for further gene expression analysis. The processed microarray data: ‘rankMatrix’ TXT file, which was downloaded from the CMap main website (https://www.broadinstitute.org/cmap/), was introduced here.

### The expression profiles of lncRNAs and genes

The data used to search coexpressed genes for lncRNAs came from the TCGA RNA-seq data. Specifically, lncRNAs expression was extracted from the TSV file ‘mitranscriptome.expr.counts’ in the MiTranscriptome database [36], and the log2 transformation was performed before correlation analysis. The mRNAs expression was extracted from the level three GExp-Gene data form the TCGA data portal. There were a total of 10 TCGA cancer types, which have more than 200 patients with both lncRNA and gene expression data available, including Breast Invasive Carcinoma (BRCA), Head and Neck Squamous Cell Carcinoma (HNSC), Kidney Renal Clear Cell Carcinoma (KIRC), Brain Lower Grade Glioma (LGG), Lung Adenocarcinoma (LUAD), Lung Squamous Cell Carcinoma (LUSC), Ovarian Serous Cystadenocarcinoma (OV), Prostate Adenocarcinoma (PRAD), Thyroid Carcinoma (THCA), and Skin Cutaneous Melanoma (SKCM) (S1 Table).

### The ALACD model

**Fig 1:**
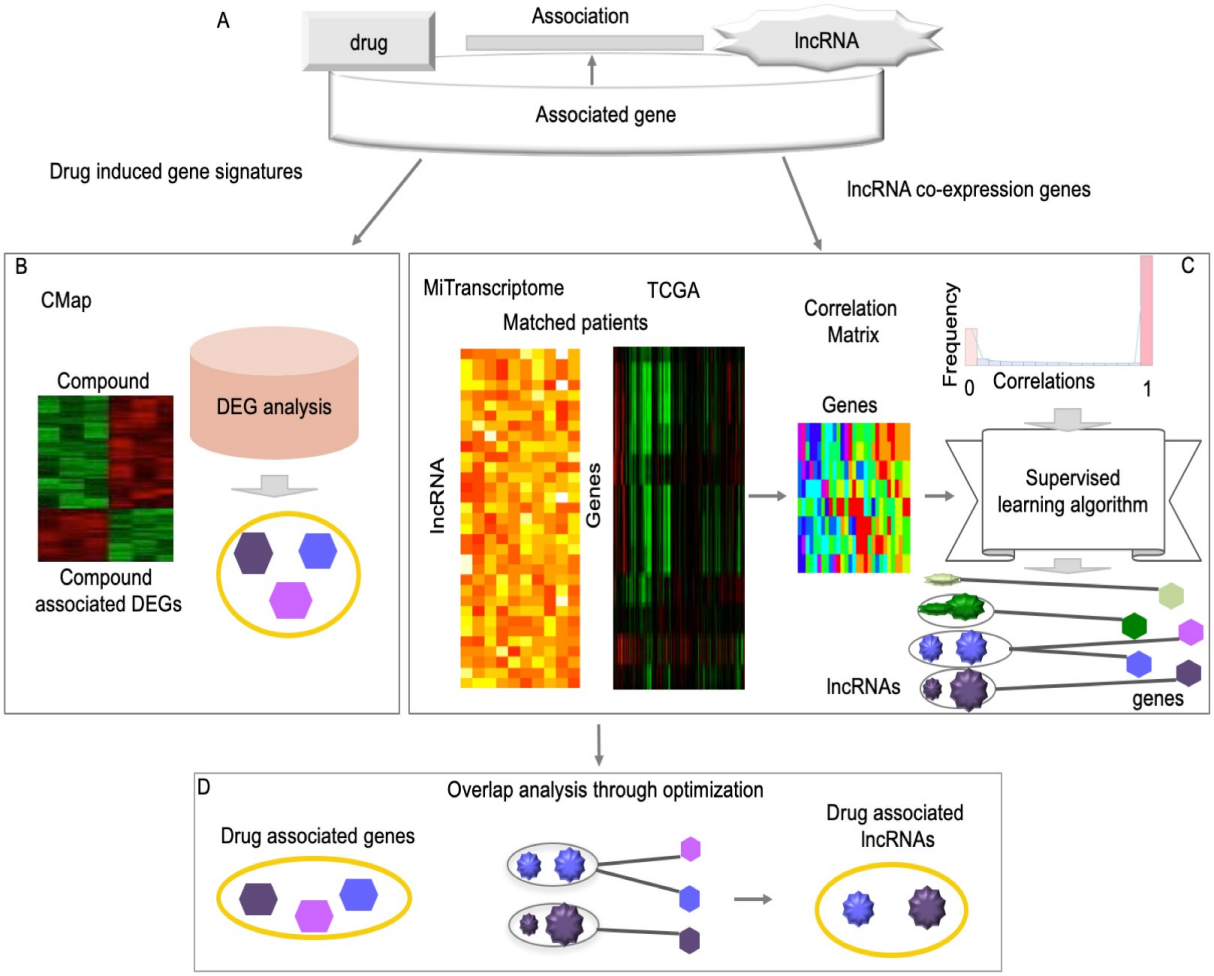
The flow-chart of ALACD. A: Association of lncRNAs with drugs via their target genes to better understand the mechanism of lncRNAs. B: Identification of drug-associated genes through gene analysis. The genes exhibiting significantly different expression levels before/after drug treatment were identified as drug-associated genes. C: Identification of lncRNA-associated genes through detecting coexpressed genes. It first constructed the initial lncRNA-gene relationships by calculating their expression correlations. Then, by ranking the whole correlation coefficients, the close and poor relationships were selected to train a supervised learning model, and the lncRNA closely associated genes were identified through this supervised learning model. The size of circle indicates the predicted score for associations between lncRNAs and genes, and the larger size means stronger association. D: Identification of anti-cancer drug associated lncRNAs through optimized associating drug’s gene signatures with their closely related genes.

We designed a computational model, named ALACD, to associate lncRNAs with drugs through their associated genes (Fig 1A). It first defined drug-associated genes by examining drug-induced gene-expression signatures (Fig 1B), and defined lncRNA-associated gene as coexpressed genes. Due to lack of known lncRNA target genes, the coexpressed genes were chosen as the lncRNA-associated genes. To further extend the coverage, the coexpressed genes were augmented by the supervised learning algorithm (Fig 1C). That is, we imputed the missing lncRNA-gene association through this supervised learning algorithm. It finally proposed an optimization model algorithm to search the optimal genes that were closely relate with both drugs and lncRNAs, and used them to associate lncRNAs with drugs (Fig 1D). More details are included in the following section.

#### Drug-associated genes: Drug induced gene signatures

The gene-expression signatures for a given drug, provided in CMap, were chosen to represent the drug associated genes. They were obtained through identification of genes showing significant differential expression levels before and after drug treatment. The coefficients indicating the relationships between the genes and a given drug (*n*_*G*_ is the number of genes) were calculated as follows:

$$\begin{array}{c} c_{i}=\text{sign}\left(logFC\right)\frac{sig_{i}-\mu }{\nu -\mu }, \end{array}$$ (1)

where *logFC* is the log transformation of fold change (FC), and sign(*logFC*) will be +1, if *logFC* larger than zero, will be -1 if *logFC* less than zero, and will be zero if FC equal to one, *sig*_*i*_ = |*logFC*| × (−log(*Pvalue*_*i*_)), *μ* and *ν* are the minimum and maximum of *sig*_*i*_, *i* ∈ {1, ⋯, *n*_*G*_}, respectively, *Pvalue*_*i*_ was Benjamini adjusted p-value and obtained by expression analysis via ‘limFit’ function in R ‘limma’ package [36]. The Eq (1) means that gene-expression signatures for CMap anti-cancer drug were those genes with absolute coefficient close to one.

#### LncRNA-associated genes: Enhanced lncRNA coexpressed genes

The genes that are coexpressed with lncRNA were defined as lncRNA-associated genes. However, only a small fraction of lncRNAs and genes are coexpressed supported by the correlation analysis (with high Pearson correlation coefficients (PCCs)). Taking the TCGA squamous cell lung cancer data as an example, only 323 pairs of lncRNAs and genes were identified to be coexpressed with high confidence (PCCs larger than 0.7), while there were a total of 10,000 possible pairs of lncRNAs and genes. To extend the range of lncRNA’s coexpressed genes, a supervised learning method was introduced. Specifically, we collected lncRNA-gene pairs with high PCCs and close to zero PCCs as training positives and negatives, respectively (S2 Fig), which were applied to train a supervised learning model (SVM classification model) by concatenating the gene and lncRNA expression levels. The rest of the possible lncRNA-gene pairs were treated as the testing data and ready for prediction via that SVM classification model. Through the supervised learning model, the associations between lncRNAs and genes were represented as the SVM score with values ranging from 0 to 1, where a strong association would have an SVM score close to one, and a weak association would be represented by an SVM score close to zero.

#### Bilevel optimization to associate drug and lncRNA

Once we obtained the associated genes for both anti-cancer drugs and lncRNAs, we could then associate them through detecting the optimum overlaping genes. To this end, an optimization algorithm was developed. Specifically, for a given drug, the correlation scores for lncRNAs were obtained by the following bilevel convex programming problem, where the upper problem, is constrained by the optimization of the lower problem:

$$\underset{d,\alpha^{*}}{\text{min}}\sum_{i=1}^{n_{G}}\left|c_{i}\right|\sum_{j=1}^{n_{L}}(\text{sign}\left(c_{i}\right)(\sum_{t=1}^{n_{T}}\alpha_{t}^{*}y_{t}K\left(x_{t},x\right)+b^{*})-d_{j}{)}^{2},$$ (2)

$$s.t.\;\;\alpha^{*}=\text{arg}\;\underset{\alpha }{\text{min}}\frac{1}{2}\sum_{p=1}^{n_{T}}\sum_{q=1}^{n_{T}}y_{p}y_{q}\alpha_{p}\alpha_{q}K\left(x_{p},x_{q}\right)-\sum_{n=1}^{n_{T}}\alpha_{n},$$ (3)

$$\begin{array}{c} \sum_{n=1}^{n_{T}}y_{n}\alpha_{n}=0,0⩽\alpha_{n}⩽C,\;n=1,\cdots ,n_{T}, \end{array}$$ (4)

$$\begin{array}{c} \exists s\in \left\{1,\cdots ,n_{T}\right\},\alpha_{s}^{*}\in \left(0,C\right),b^{*}=y_{s}-\sum_{n=1}^{n_{T}}y_{n}\alpha_{n}^{*}K\left(x_{n},x_{s}\right), \end{array}$$ (5)

where *n*_*T*_ is number of lncRNA-gene pair used for training, **x** is a pair of a lncRNA and a gene, which was represented through concatenation of lncRNA and gene expression profile; *y*_*t*_ = 1, when the *t*th pair of lncRNA and gene is positive (PCC larger than 0.7 in BRCA patients), *y*_*t*_ = −1, when the *t*th pair of lncRNA and gene is negative (PCC is zero in BRCA patients). The enhanced associations between lncRNAs and genes were learned by solving the lower problem (SVM standard classification model), and the associations between lncRNAs and anti-cancer drugs were learned by solving the upper problem. The rationale of the upper optimization model is to perform the overlapping analysis between the drug’s gene-expression signatures and the lncRNA’s closely associated genes. The optimization procedure forces drug linkage with the lncRNAs, which were closely associated with drug-induced gene signatures. In addition, a rank score (RS) was applied to ensure that the above optimization procedure could reveal as many as possible genes associated with both drugs and lncRNAs: $RS=\frac{f_{b}+0.5\times f_{w}}{N}\times 100$, where *f*_*b*_ is number of lncRNAs with fewer overlapping genes than the predicted one, *f*_*w*_ iis the number of lncRNAs with overlapping genes equal to the predicted one, and *N* is number of all lncRNAs.

### Model implementation and survival analysis

The above bilevel optimization problem was solved first by solving the lower problem, and then by finding the optimum solution for the upper problem. The lower problem was actually the SVM standard classification model, which was performed by using LibSVM in ‘e1071’ R package [38]. The penalty parameter and the RBF kernel parameter were optimized by the grid search approach with 3-fold cross-validation. The performance of this SVM model was evaluated through 5-fold cross-validation. The evaluation criteria, AUC (area under the curve), receiver operating characteristic (ROC) curve [39], AUPR (area under the precision-recall) curve [40], accuracy (ACC), sensitivity (Sn), specificity (Sp), precision (Pre), and F-measure (geometric mean of Sn and Sp), were used to assess the performance of the supervised learning model.

The upper optimization problem is convex quadratic programming, which was implemented through the ‘nlm’ function in the R programming language with zero as the initial points. Through solving the optimization problem, for each drug, we obtained the correlation scores for all lncRNAs. By ranking them in descending order, the top five lncRNAs with p-value less than 0.05 were considered as drug-associated lncRNAs. The p-value displayed the specificity of the linkage of the lncRNA with the given drug, and was calculated through the frequency of the lncRNAs in the top score lists of all CMap drugs.

To display the usefulness of the identified lncRNAs in cancer prognosis, the Kaplan-Meier survival analysis [41] was introduced. Specifically, the survival information was collected from the TCGA clinical data, and the patients were divided into two classes according to the expression level of a lncRNA: patients with a high expression level (higher than 6), and patients with a low expression level (lower than 2). The thresholds for high and low expression levels were determined by the distribution of lncRNA expression in cancer patients (S3 Fig). Then, the correlation analysis for patient survival on those two classes of patients was performed via ‘survival’ R package.

### Validations based on the lncRNA2Target database

To demonstrate the effectiveness of the augmented model, the SVM model was applied to the lncRNA2Target (version 1) lncRNA-gene association data [42]. The lncRNA2Target (version 1) deposits human and mouse lncRNA-to-target genes based on lncRNA knockdown or overexpression experiments. The expression data for lncRNAs and genes in lncRNA2Target (version 1) also came from MiTranscriptiome and TCGA data portal, respectively. The predicted score (SVM score), which indicates how strong the relationship between the lncRNA-gene pairs is, is displayed.

### Functional and pathway enrichment analysis

To display the role of gene signatures bridging drugs and lncRNAs, functional enrichment analysis was performed through using GO terms and KEGG pathway annotations via DAVID Bioinformatics Resources. The enrichment terms with Benjamini adjusted p-value less than 0.01 was reported, and for those genes without such enrichment terms, the GO molecular functions (MFs) and KEGG pathways shared by over 60% of the genes were reported.

## Results

### The supervised learning algorithm increases the coverage of lncRNA-associated genes

**Fig 2:**
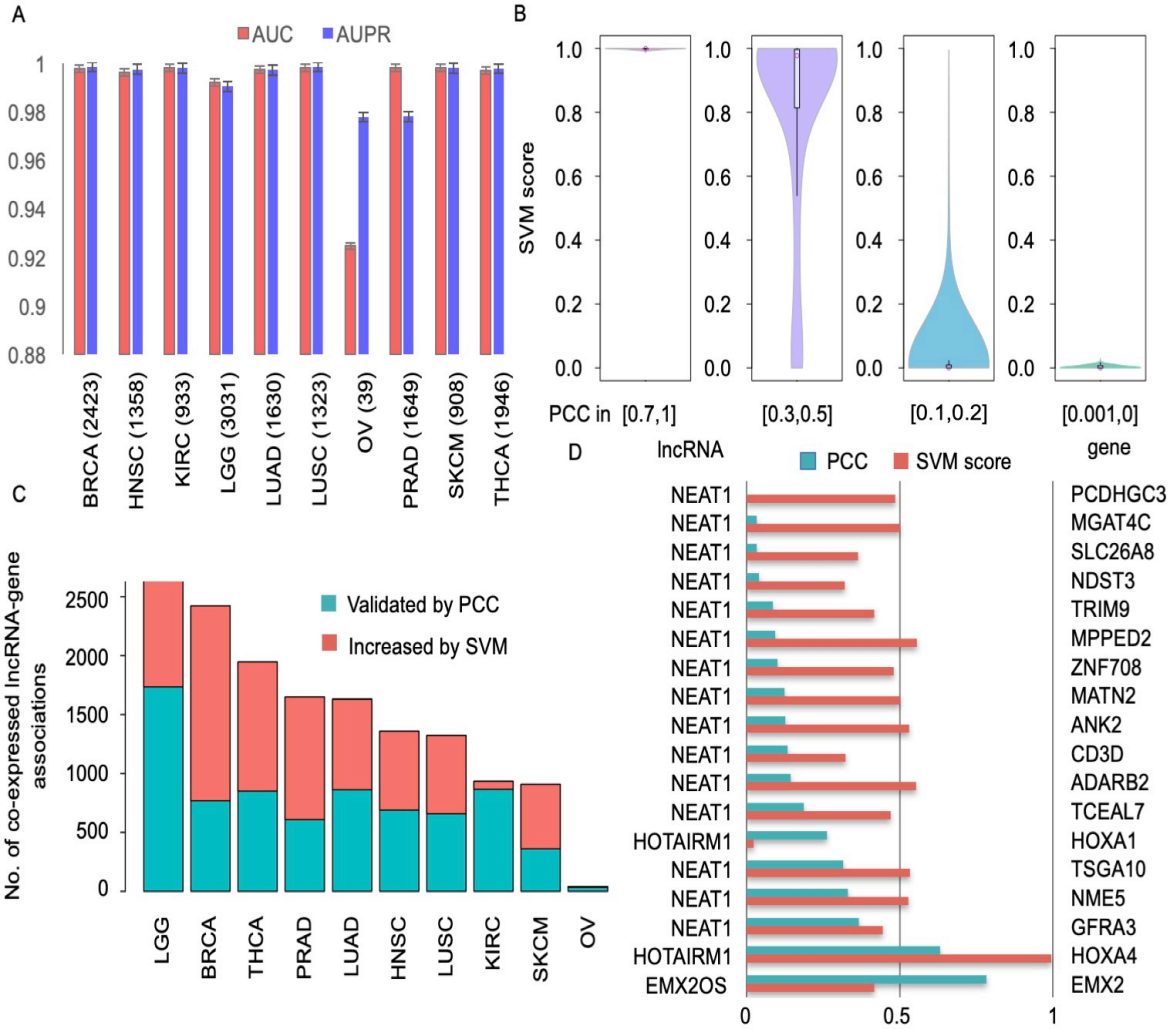
The performance of the SVM model in estimating correlation coefficients and its advantage. A: The AUC and AUPR of ten cancer types, and the number of positives in each type of cancer is indicated. B: The boxplot displays the correlation between SVM score and PCCs. The four groups are generated according to the value of PCCs. C: The coexpressed lncRNA-gene associations expanded by the SVM model. The green bar indicates the coexpressed lncRNA-gene associations validated by PCCs (larger than 0.5), and the red bar indicates the increased coexpressed lncRNA-gene associations from the SVM model with SVM scores larger than 0.9. D: The independent dataset validation: lncRNA2Target (version 1), and the PCCs and SVM scores for 18 lncRNA-gene pairs are displayed by green and red bars, respectively.

The supervised learning algorithm was introduced to augment the lncRNAs’ coexpressed genes. To evaluate its performance, a two-step validation process was performed. First, we asked whether it could simulate the PCCs effectively. Except for OV data with only 39 positives for training, close to one evaluation criteria were obtained for all other cancer types (Fig 2A and S4 Fig). Furthermore, the predicted scores with value close to one signified close correlation, and close to zero denoted weak correlation (Fig 2B), which indicated that the supervised model could simulate coexpression relationships quite well.

Then, we asked whether the supervised model could extend the searching space for lncRNA-associated genes. The cross-validation results showed that 7.2% to 68.3% of lncRNA-gene pairs with PCCs larger than 0.5 and smaller than 0.7 (larger than 0.3 and smaller than 0.5 for those PCCs larger than 0.5 as positives) had a predicted score larger than 0.9 (Fig 2C and S1 Table), while there were more than ten thousand lncRNA-gene pairs with PCC larger than 0.5 and smaller than 0.7 (larger than 0.3 and smaller than 0.5 for those PCCs larger than 0.5 as positives). This means that the supervised model could increase the coverage of lncRNA-associated genes by a factor of at least 1e+3. To further validate this, we introduced the lncRNA target gene database, LncRNA2Target (version 1), as independent test data. After collecting the matched expression information from MiTranscriptome and TCGA for human lncRNA-gene associations in LncRNA2Target (version 1), 18 associations remained (S2 Table). Among all 18 associations, only two of them had PCCs larger than 0.5 (*EMX2OS-EMX2, HOTAIRM1-HOXA4*), and except for two low-throughput lncRNA-gene associations (*HOTAIRM1-HOXA1, EMX2OS-EMX2*), the SVM scores were much higher than the PCCs for the remaining 16 lncRNA-gene associations (Fig 2D). All these findings indicate that the supervised learning algorithm could not only increase the coverage of lncRNA-associated genes, but also detect potential lncRNA target genes. The supervised model could increase the number of lncRNA-associated genes because it borrows information about the associations from lncRNAs or genes that have similar expression patterns. That is, the given lncRNA-gene association could be uncovered by searching for lncRNAs that have expression pattern similar to those of the given lncRNA (S5A and S5C Fig), or for genes that have expression pattern similar to those of the given gene (S5B and S5D Fig).

### The associations between lncRNAs and anti-cancer drugs in TCGA tumors

**Fig 3:**
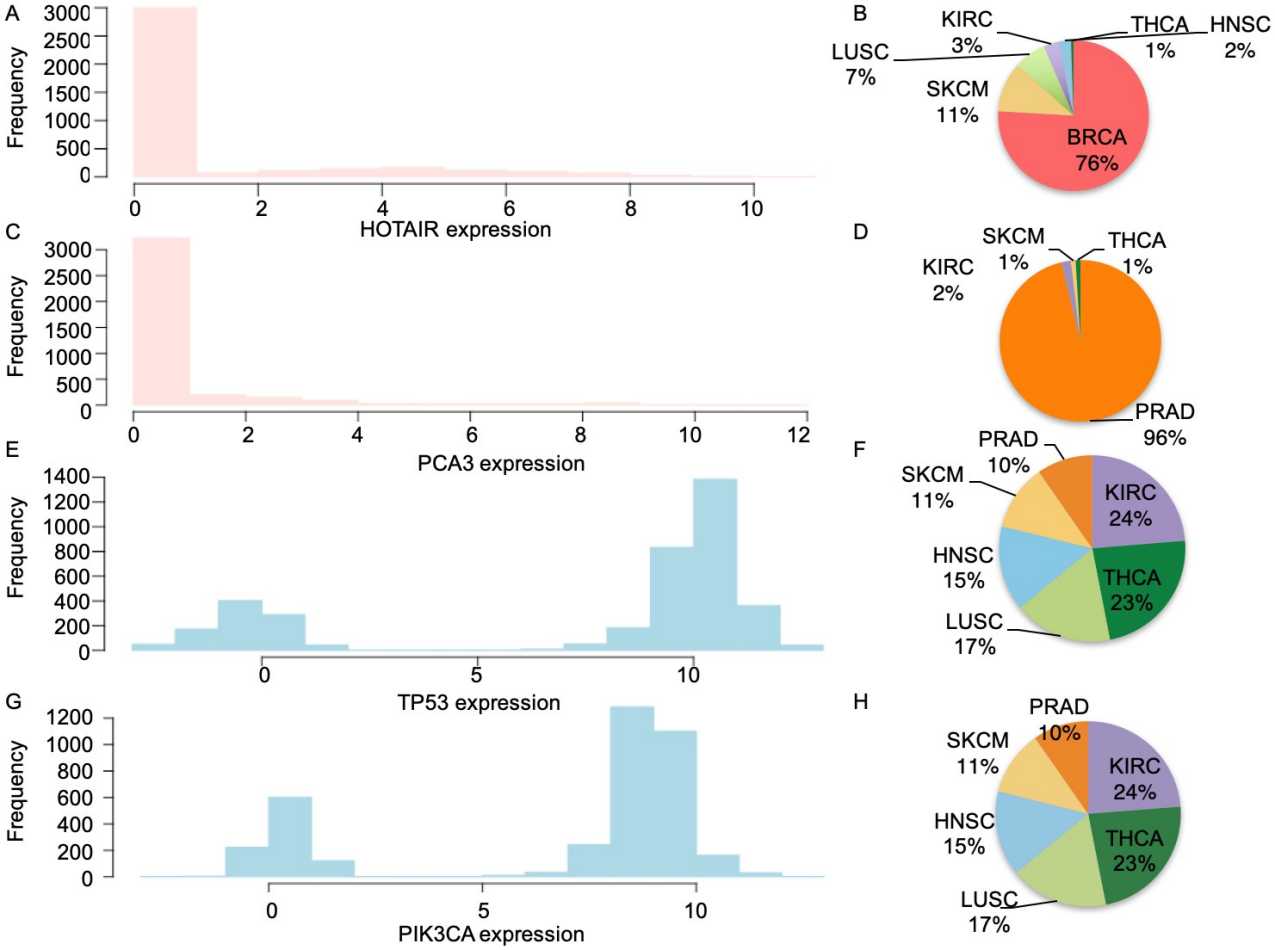
The specificity of lncRNAs indicates their unique properties. The expression pattern of lncRNAs (A,C) and genes (E,G) in all tumor patients. The piecharts show the tumor types in which the lncRNAs (B,D) and genes (F,H) were highly expressed (expression levels higher than 4 for lncRNAs and larger than 6 for genes).

The ALACD model was applied to ten types of TCGA cancer patients. We summarized the predictions on all ten types of cancer and addressed the following three observations. First, for a specific tumor type, a given drug was associated with more than one lncRNA (S6A Fig), and a lncRNA was connected with more than one drug (S6B Fig). Second, for a specific drug, associations were observed with unique lncRNAs in different cancer types. That is, drugs did not share lncRNAs across different cancer types. Taking aspirin (acetylsalicylic acid) as an example, the ALACD model identified 10 unique lncRNAs for 10 distinct types of cancer (S6C Fig). Third, when summarizing all drug-associated lncRNAs in a certain cancer type, we found that different types of cancer had its unique lncRNAs, which increased the potential identification of specific candidate therapeutic targets. Only breast cancer and lung adenocarcinoma were associated with the same lncRNAs, which were *CAT354* and *LINC00665.6*, while prostate and thyroid cancers shared the lncRNA *LINC00958.9* (S6D Fig). The potential reason why either drug or disease did not share lncRNA targets may be because lncRNAs are specifically highly expressed in certain types of cancer. For instance, the breast cancer-related lncRNA *HOTAIR* is silent in most cancer patients (Fig 3A), and is highly expressed in only a few of tumor types, most of which are breast cancers (Fig 3B). The prostate cancer related lncRNA *PCA3* is silent in most cancer patients (Fig 3C), and is highly expressed in 96% of prostate cancers (Fig 3D). While the cancer genes, such as *TP53* and *PIK3CA*, have significantly different distribution than lncRNAs (p-value less than1e-5 by KS-test). They are highly expressed in most cancer patients, and silent in only a few of them (Fig 3E ∼ 3H).

### The associations between lncRNAs and anti-cancer drugs in an individual tumor type facilitate understanding of the mechanism of lncRNAs and their roles in cancer

**Fig 4:**
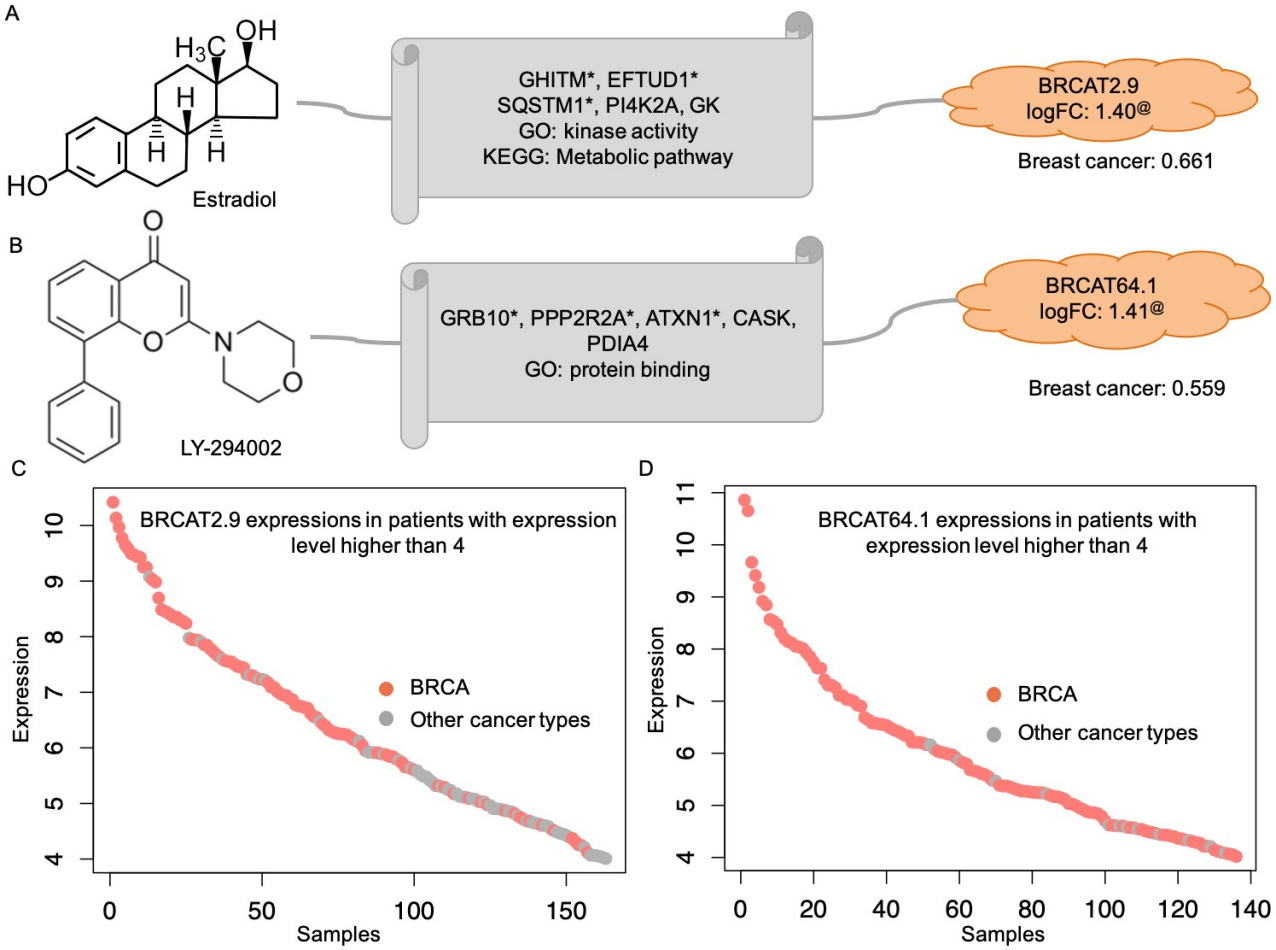
Representative prediction examples in BRCA. A, B: Two prediction examples in BRCA. The linkage of associated genes with breast cancer is suggested by the literature (*), and the linkage of lncRNA with breast cancer is exhibited by the confidence score. The differential expression of lncRNAs in breast cancer is shown by the logFC and adjusted Benjamin p-value less than 0.001 (@). C, D: The specificity of lncRNA in breast cancer is displayed by the expression profile of lncRNA in patients with expression level larger than 4.

The drug-associated lncRNAs in cancer patients that were identified through the ALACD model indicate their unique properties in different tumor types. Therefore, we further analyzed the lncRNAs in individual tumor types to understand the role of lncRNAs in each particular type of cancer. The close relationship of lncRNAs with cancer was supported through three phases: the literature evidence for the linkage between associated genes and cancer types, the confidence score for relationships between lncRNAs and types of cancer collected from MiTranscriptome, and the expression specificity of lncRNAs. The linkage between lncRNAs and anti-cancer drugs was established through the associated genes, and the annotation of these genes helped us understand the function of lncRNAs in cancer. Thus, functional and pathway enrichment analysis were performed and enriched GO terms and KEGG pathways are shown in S3∼S12 Tables. For instance, estradiol, a form of estrogen, was associated with *BRCAT2.9* through the ALACD model (Fig 4A), which was specifically expressed in BRCA patients (Fig 4C). Three out of the five genes associated with estradiol and *BRCAT2.9* were linked to breast cancer according to the literature [43–45], and they shared kinase activity, suggesting that the molecular function of *BRCAT2.9* is involved in metabolism. In addition, MiTranscriptome suggested the association of *BRCAT2.9* with breast cancer with a confidence score larger than 0.6. All these results support the linkage of *BRCAT2.9* with breast cancer. LY294002, which was reported to be related to breast cancer cell apoptosis [46], was associated with *BRCAT64.1* through the ALACD model (Fig 4B). Three out of the five genes that were associated with LY294002 and *BRCAT64.1* were linked to breast cancer, as supported by the literature [47–49]. In addition, *BRCAT64.1* was associated with breast cancer in MiTranscriptome with a confidence score larger than 0.5, and was specifically expressed in breast cancer (Fig 4D). These results suggested the important role of *BRCAT64.1* in breast cancer.

The optimization program (Eqs (2) ∼ (5)) indicated that ALACD could uncover the associations between lncRNAs and genes through revealing as many associated genes as possible. To demonstrate that, a rank score (RS) was defined (see Methods). The rank scores that were close to one hundred (S3∼S12 Tables) suggested that there were few of lncRNAs that shared a larger number of associated genes with a given drug than the predicted one, which supports the close relationship between drugs and predicted lncRNAs. That is, ALACD suggests drug-associated lncRNAs through finding as many genes as possible that are closely associated with them. The functions of these genes provide a way to understand the functions of lncRNAs in cancer.

To further understand the role of lncRNAs in cancer, the expression variability of lncRNAs in cancer patients was evaluated. That is, we collected the data for lncRNA expression in TCGA normal samples, and compared them with the expression in TCGA tumor samples. As a result, approxinately 70% (75/108) of the predicted lncRNAs had absolute logFC values larger than 1.2, and Benjamin p-values less than 0.001 (S3∼S12 Tables). All these results emphasize the importance of the predicted lncRNAs in cancer, and these identified lncRNAs may provide great opportunities for developing novel target-specific therapeutics.

### Specificity of lncRNAs and survival analysis indicate alternative choices for cancer treatment

The prediction results suggest that ALACD provides information for an alternative choice for cancer treatment. This is because, through ALACD, we can identify potential lncRNA targets, that participate in biological processes similar to those controlled by anti-cancer drugs. To validate this, among all our predictions, we focused on the lncRNAs, which were differentially and specifically expressed in a certain tumor type. The associated drugs were reported to relate with the corresponding type of cancer, and the associated genes were linked to the tumor type, as supported either by the literature or by their expression profile. The specificity of lncRNAs was illustrated by their expression profiles in cancer patients with high expression levels (expression larger than 4), and if lncRNA is designed as specific to a tumor type, that means this cancer type had more than 50% of patients with expression higher than 4. As a result, 14 lncRNAs met those criteria and have great potential as therapeutic targets (S13 Table). The expression profiles of lncRNAs in caner patients with high expression (S7 Fig) and the expression variation analysis indicate that the predicted lncRNAs were specifically and differentially expressed in their associated cancer types, and the close relationships between their associated genes and the diseases and published reports (supplementary information), indicate the close relationship of lncRNAs and cancer.

**Fig 5:**
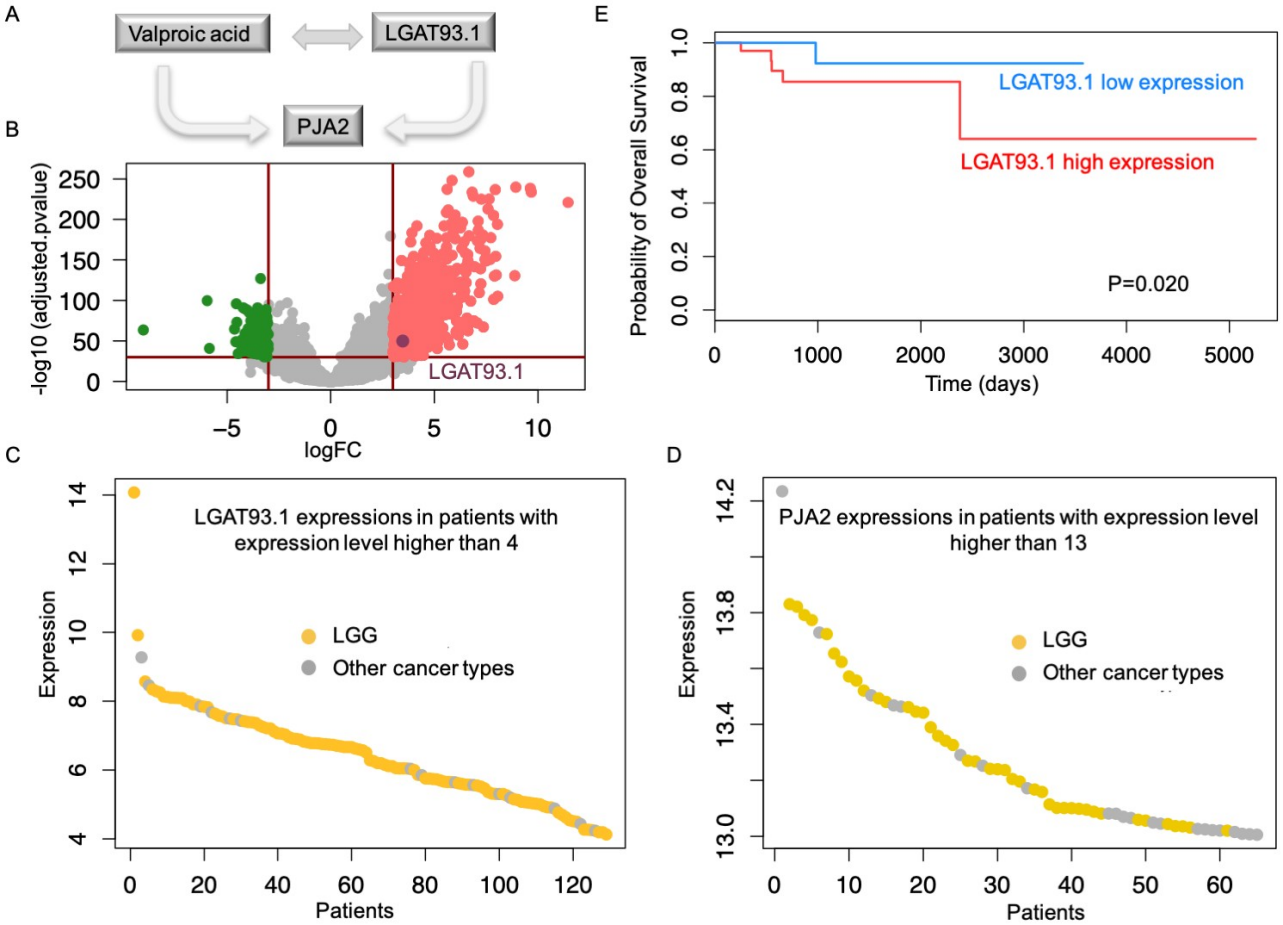
Representative examples of candidates that are worthy for further clinical study. A: The lncRNA *LGAT93.1* was predicted as an alternative anti-cancer drug target for the drug valproic acid. B: The volcano plot for lncRNAs when comparing expression in LGG tumor samples with normal samples, and the lncRNA *LGAT93.1* is highlighted by a darkpink circle. C: The expression of *LGAT93.1* in patients with expression levels larger than 4. D: The expression of the associated gene *PJA2* in patients with expression values larger than 13. E: The significantly strong correlation of *LGAT93.1* expression with LGG patient survival suggests a prognostic biomarker for LGG treatment.

To further demonstrate the usefulness of above predicted lncRNAs in cancer treatment, prognosis validation was implemented through Kaplan-Meier survival analysis. As a result, we identified 9 lncRNAs that correlated with patient survival (S14 Table and S8 Fig) among above 14 specifically and differentially expressed lncRNAs. In detail, these lncRNAs were not only specifically and differentially expressed in their associated cancer types, but were also correlated with patient survival. In addition, their associated genes were closely linked to cancer, and this was supported by either the literature or their expression profiles (S14 Table). Furthermore, among all nine predictions, four of them strongly correlated with patient survival (p-value less than 0.05), and two of them were associated with cancer, with which the associated drug had not been previously reported to be linked (*HNCAT60* and *HNCAT30.1*). For instance, the drug fluphenazine was linked to myeloma according to the literature reports [50], but had not been previously used for the treatment of patients with HNSC. Therefore, *HNCAT60*, which displayed specific and differential expression in HNSC cancer patients and was closely related to patient survival (S8 Fig), could be a great alternative target for the treatment of HNSC. LncRNA *LGAT93.1* was associated with the drug valproic acid (Fig 5A), a fatty acid with anticonvulsant properties, and was linked to LGG in MiTranscriptome with confidence score of 0.621. In addition, *LGAT93.1* was specifically and differentially expressed in LGG (Fig 5B and 5C), and the expression of its associated gene *PJA2* was specifically highly expressed in LGG patients (Fig 5D). Furthermore, *LGAT93.1* significantly related to LGG patient survival (p-value less than 0.1, Fig 5E), that is, patient subtypes with *LGAT93.1* exhibited significantly different survival rates. Thus, targeting *LGAT93.1* provides a novel therapeutic choice for the treatment of LGG, which has the potential to improve patient survival. In the future, further validations could test cancer cell activity after silencing *LGAT93.1* to assess the variations in the expression levels of the associated gene *PJA2*, which could help to reveal the underlying role of *LGAT93.1* in LGG. Collectively, the results indicate that the ALACD model provides valuable information about alternative targets for small molecules in cancer treatment, and based on similar regulatory functions, identified lncRNAs exhibit novel prognostic evidence in clinical application.

## Discussion

The involvement of lncRNAs in the development of complex diseases, including cancer, indicates the potential usage of lncRNAs for the development of novel treatment agents. Several features of lncRNAs render the possibility of lncRNAs as therapeutic targets and some lncRNA therapeutics are currently being investigated. In this paper, we propose a computational method, the ALACD model, to associate lncRNAs with anti-cancer drugs. Through bilevel optimization, we provide additional information to increase understanding of the mechanism of action of lncRNAs and their roles in disease. In addition, with their specificity and differential expression in cancer patients, and strong relationship with patient survival, the lncRNAs identified by the ALACD model could be treated as alternative agents for their associated drugs. Here, we attempted to provide three criteria for candidate lncRNAs (S14 Table) that involve the regulation in cancer and are worthy for further clinical studies. That is, first, the lncRNAs have to be specifically and differentially expressed in a certain type of cancer patients; second, the expression of lncRNA has to be associated with patient survival; third, the associated genes have to be disease-related genes.

The methodology of ALACD indicates that it can adapt to other types of data sources. Specificity, if including the drug response data from TCGA, the ALACD model will generate the lncRNAs that associate with drug sensitivity/resistance. This is distinct form determining the molecular mechanism. Here, we would like to identify the cancer-associated lncRNAs that perform regulatory roles that are similar to anti-cancer drugs in cancer patients; if using drug response data in cancer patients, this method will generate lncRNAs that are related to drug inhibition effects in cancer patients, and those lncRNAs could be the genomic signatures for cancer sensitivity. To check how our result was affected by using drug response data in TCGA to determine the drug-associated lncRNAs, we ran our ALACD model with TCGA drug response data instead of CMap data. That is, we defined the drug-induced gene signatures as the genes showing significantly different expression levels in patients who responded or did not respond to that drug. Specifically, we collected the clinical drug responses of TCGA cancer patients from previous work [51]. As in [51], the clinical responses were divided into two classes: responders (including complete response and partial response) and nonresponders (including stable disease and progressive disease). We removed patients with possible combination therapy and chemotherapy prior to surgery, and kept the response data for those drugs that had more than 10 responders and 10 nonresponders. As a result, the response data for 10 drugs in 943 patients with available expression data was introduced for validation. The predicted drug-lncRNA associations are listed in S15 Table (the MiTranscriptome-suggested cancer type and confidence score are also shown). From that table, we could see that, most of the predictions (77%) were supported by the MiTranscriptome database. Different from the previous work ([17–20]) aimed at revealing the lncRNAs associated with drug sensitivity/resistance through gene expression analysis, ALACD, uses bilevel optimization procedure to link lncRNAs with drug sensitivity/resistance through their associated genes.

The lack of conversion across the species limits the study of function for lncRNAs by transferring the annotation form validated lncRNA to a newly discovered one, and characterizing the function of lncRNAs through biological experiments is costly and time-consuming. Currently, there are some curated databases depositing characterized lncRNAs, such as LNCipedia [52], NONCODE [53], lncRNAdb [54], and lncRNAWiki [55]. Moreover, the lncRNA2Disease database [56] has deposited more than 1000 lncRNA-disease entries and 475 lncRNA interaction entries, including 321 lncRNAs and 221 diseases from about 500 publications. These valuable data resources provide a way to build a machine learning-based computational model to learn the potential rule of lncRNAs in disease. While much more well-defined lncRNA-disease associations are needed to increase the generalization of the machine learning model. In addition, the lncRNA2Disease only composed of human associations, and the low conservation limits the study of lncRNA function by transferring knowledge from known species to unknown ones. However, researchers still developed some computational models to address this topic, and they could be divided into three types: one is identifying the differentially expressed lncRNAs to link with cancer; another is revealing lncRNAs whose expression would be associated with drug sensitivity/resistance; the last is borrowing the information from some mediator (such as miRNA) to associate lncRNAs with cancer.

The ALACD model differs from existing models in the following ways: First, the coexpressed genes were introduced by both previous works ([16, 57–59] and ALACD. However, to impute the missing lncRNA-gene coexpressed association, a supervised learning algorithm was introduced in ALACD. Second, unlike previous works that generated the lncRNA signatures through identifying the lncRNAs that display significantly different expression levels before/after small molecule treatment, ALACD uses an optimization procedure to link lncRNAs with drugs through their associated genes. Third, in previous works, the author either provided the cancer-related lncRNAs ([16]) or drug-lncRNA associations ([19, 20]). While, through our ALACD model, lncRNAs, the associated anti-cancer drug, and the induced gene signatures involved in the regulation of cancer, are collectively uncovered. Although the initial aim of ALACD was to associate lncRNAs with small molecules, the lncRNAs identified from the lncRNA-drug associations still exhibit significantly different expression levels in cancer patients. Moreover, the follow-up functional and molecular pathway analyses suggest the close relationships of signature genes and lncRNAs with cancer development. Importantly, patient survival information and evidence in the literature suggest that the lncRNAs and drug-lncRNA associations identified by ALACD provide an alternative choice for cancer-targeting treatment and potential prognostic biomarkers.

Many works discuss the associations between miRNAs and small molecules. Comparing with prediction of miRNA-small molecule association [60, 61] there are some challenges in predicting lncRNA-small molecule associations. First, comparing with lncRNA, miRNA is a type of noncoding RNA that is relatively well studied, and there is some existing knowledge to help build the similarity network about miRNAs, such as miRNA-disease associations [22–24], miRNA-mRNA interactions [62, 63], etc. Second, compared with miRNA and other RNA molecules, lncRNAs have shown low conservation across species, low expression levels in cells, and high tissue- or condition-specificity. Thus, it is quite challenging in transferring the knowledge from characterized lncRNAs. Third, lncRNAs with similar functions often lack linear sequence homology, and the complicated regulatory function of lncRNAs challenges the development of a predictive model.

The ideal associated genes for lncRNAs are genes that are regulated by lncRNAs, because identification of genes that are also associated with drugs could help ALACD generate lncRNAs, that are involved in cancer regulation by actually regulating drug-induced gene signatures. Recently, researchers have developed some curated databases, which deposit experimentally validated lncRNA target genes, such as LongHorn [64], EVLncRNAs [65], RISE [66] etc. These valuable data sources are certainly ready to be incorporated into our ALACD model. It would support the interpretation of the current results and allow us to further understand the role of lncRNAs in cancer.

## Supporting information

S1 Fig CMap data pool and the histogram of the number of treatment instances with respect to CMap drugs. Only drugs with more than 10 treatment instances were retained for further analysis.
(TIF)

S2 Fig The histogram of correlation coefficients between lncRNAs and genes in BRCA patients. The pairs of lncRNAs and genes with coefficients larger than 0.7 and close to zero (less than 0.000002) were selected as positives and negatives, respectively, to train SVM classifier.
(TIF)

S3 Fig The histogram of BRCAT47 expression in BRCA patients. The threshold for low and high expression levels are shown by red and green bars, respectively.
(TIF)

S4 Fig SVM performance on all ten cancer types evaluated with various evaluation criteria. (TIF)

S5 Fig The promising advantage of the supervised learning method. The prediction example in BRCA (A) and HNSC (B). C, The heatmap for aspirin (acetylsalicylic acid) associated lncRNAs across 10 cancer types. D, The heatmap for lncRNAs for 10 cancer types.
(TIF)

S6 Fig The predicted examples in BRCA (A) and HNSC (B). C, The heatmap for aspirin (acetylsalicylic acid) associated lncRNAs across 10 cancer types. D, The heatmap for lncRNAs for 10 cancer types.
(TIF)

S7 Fig The expression profile of lncRNAs in patients with high expression levels. (TIF)

S8 Fig Survival plots for nine predictions, which are worthy of further experimental validations. (TIF)

S1 Table The TCGA tumor types used in validation of ALACD. (DOCX)

S2 Table The correlation coefficients and predicted scores for lncRNA-gene associations in the LncRNA2Target data. The better results are highlighted in bold.
(DOCX)

S3 Table The predicted associations between lncRNAs and anti-cancer drugs in BRCA. The literature supports for associations of genes with corresponding type of cancer are suggested. Note: * adjustment p-value less than 0.001.
(DOCX)

S4 Table The predicted associations between lncRNAs and anti-cancer drugs in HNSC. The literature supports for associations of genes with corresponding type of cancer are suggested. Note: * adjustment p-value less than 0.001.
(DOCX)

S5 Table The predicted associations between lncRNAs and anti-cancer drugs in KIRC. The literature supports for associations of genes with corresponding type of cancer are suggested. Note: * adjustment p-value less than 0.001.
(DOCX)

S6 Table The predicted associations between lncRNAs and anti-cancer drugs in LGG. The literature supports for associations of genes with corresponding type of cancer are suggested. Note: * adjustment p-value less than 0.001.
(DOCX)

S7 Table The predicted associations between lncRNAs and anti-cancer drugs in LUAD. The literature supports for associations of genes with corresponding type of cancer are suggested. Note: * adjustment p-value less than 0.001.
(DOCX)

S8 Table The predicted associations between lncRNAs and anti-cancer drugs in LUSC. The literature supports for associations of genes with corresponding type of cancer are suggested. Note: * adjustment p-value less than 0.001.
(DOCX)

S9 Table The predicted associations between lncRNAs and anti-cancer drugs in OV. The literature supports for associations of genes with corresponding type of cancer are suggested. Note: * adjustment p-value less than 0.001.
(DOCX)

S10 Table The predicted associations between lncRNAs and anti-cancer drugs in PRAD. The literature supports for associations of genes with corresponding type of cancer are suggested. Note: * adjustment p-value less than 0.001.
(DOCX)

S11 Table The predicted associations between lncRNAs and anti-cancer drugs in SKCM. The literature supports for associations of genes with corresponding type of cancer are suggested. Note: * adjustment p-value less than 0.001.
(DOCX)

S12 Table The predicted associations between lncRNAs and anti-cancer drugs in THCA. The literature supports for associations of genes with corresponding type of cancer are suggested. Note: * adjustment p-value less than 0.001.
(DOCX)

S13 Table The predictions with strongpotential as therapeutic targets. (DOCX)

S14 Table The candidates that are related to patient survival are worthy for further clinical study. Note: * adjusted p-value less than 0.001; # survival p-value less than 0.25; ## survival p-value less than 0.05.
(DOCX)

S15 Table The predicted drug-lncRNA associations based on TCGA drug response data. (DOCX)
