# Supplementary figures and images for: Associating lncRNAs with small molecules via bilevel optimization reveals cancer-related lncRNAs
Source: PLoS Comput Biol. 2019 Dec 26;15(12):e1007540. doi: 10.1371/journal.pcbi.1007540 (PMC6948815; doi:10.1371/journal.pcbi.1007540)

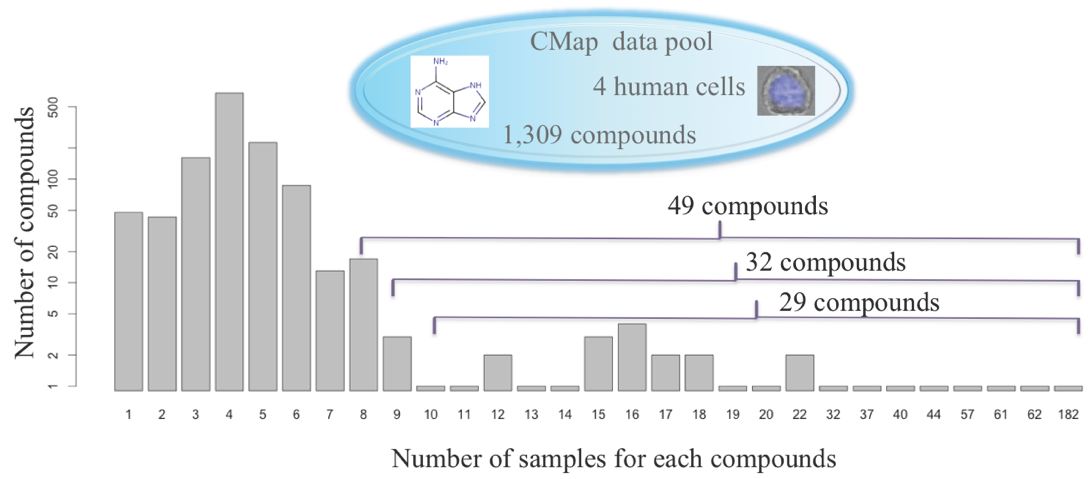

Supplement: S1 Fig — Only drugs with more than 10 treatment instances were retained for further analysis. (TIF) [file pcbi.1007540.s001.tif]

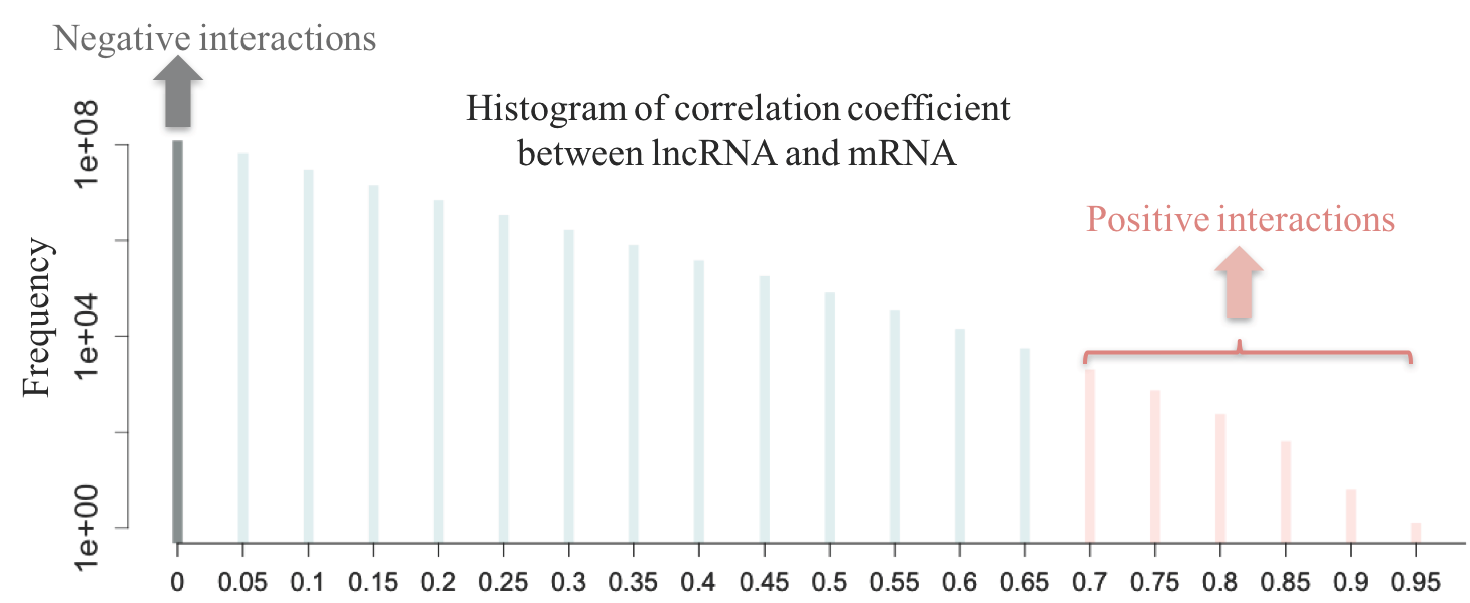

Supplement: S2 Fig — The pairs of lncRNAs and genes with coefficients larger than 0.7 and close to zero (less than 0.000002) were selected as positives and negatives, respectively, to train SVM classifier. (TIF) [file pcbi.1007540.s002.tif]

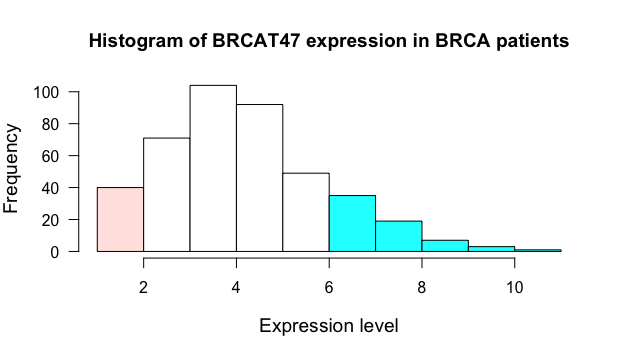

Supplement: S3 Fig — The threshold for low and high expression levels are shown by red and green bars, respectively. (TIF) [file pcbi.1007540.s003.tif]

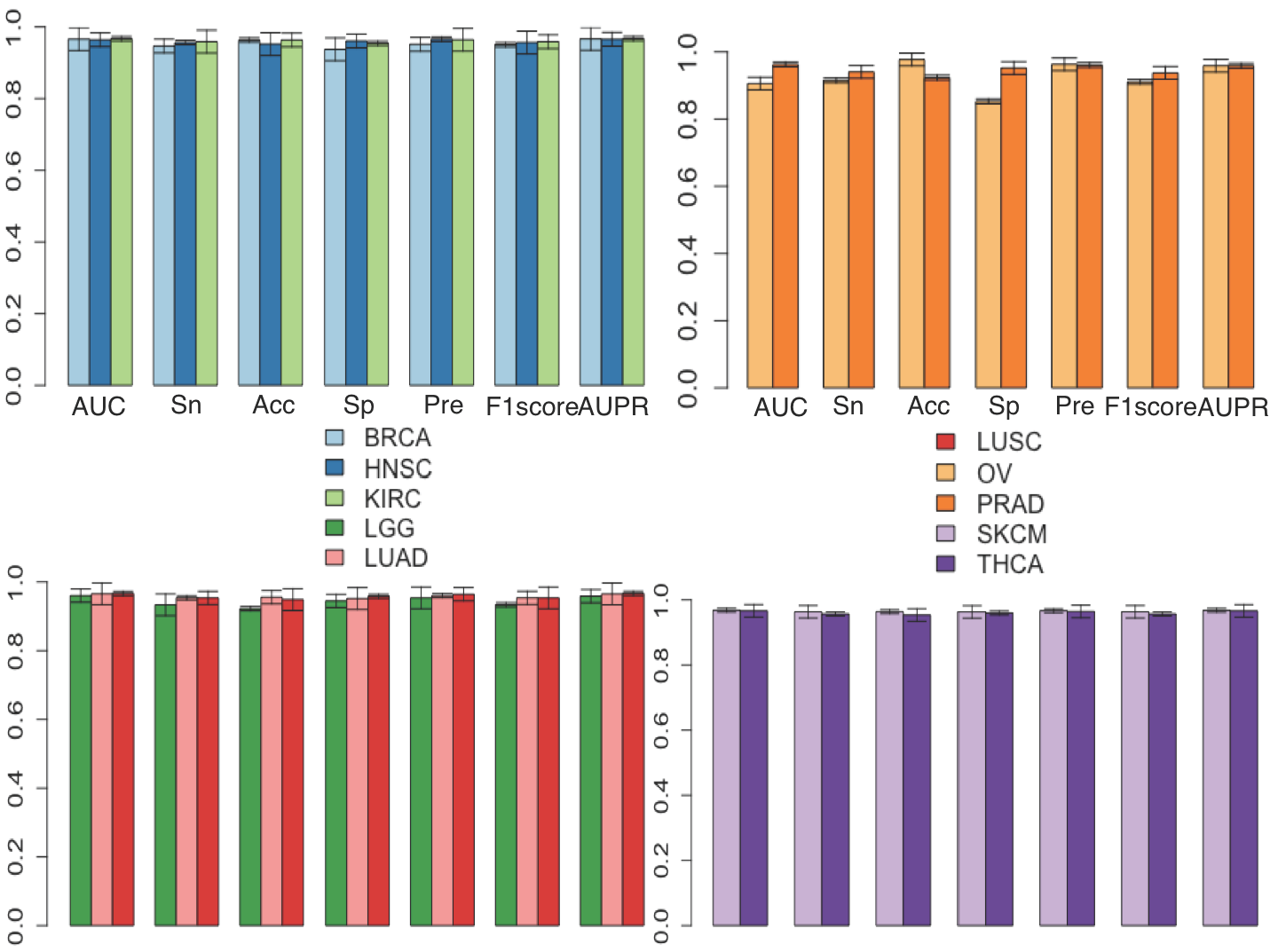

Supplement: S4 Fig — (TIF) [file pcbi.1007540.s004.tif]

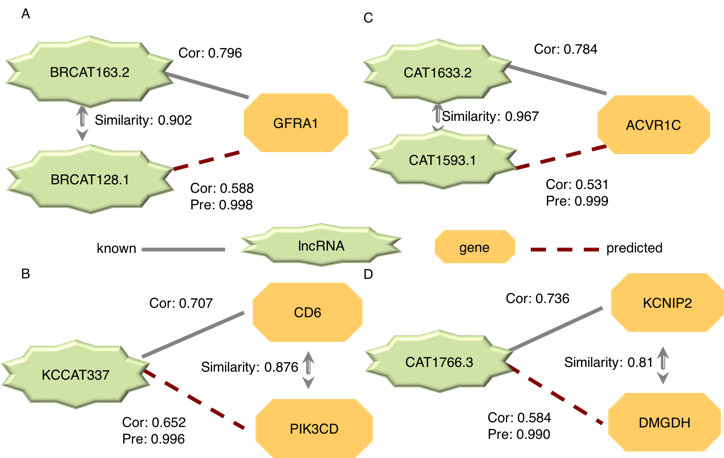

Supplement: S5 Fig — The prediction example in BRCA (A) and HNSC (B). C, The heatmap for aspirin (acetylsalicylic acid) associated lncRNAs across 10 cancer types. D, The heatmap for lncRNAs for 10 cancer types. (TIF) [file pcbi.1007540.s005.tif]

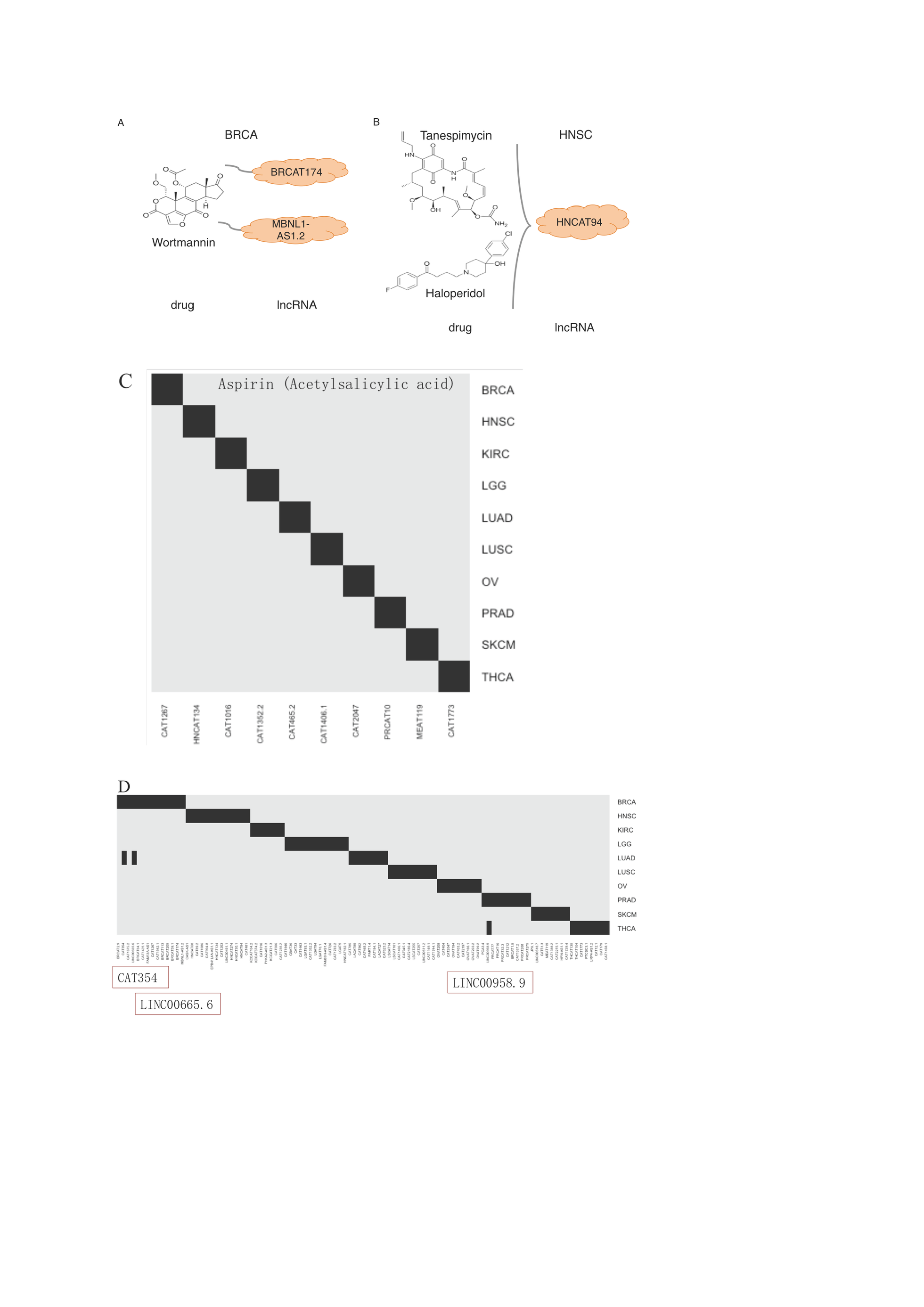

Supplement: S6 Fig — The predicted examples in BRCA (A) and HNSC (B). C, The heatmap for aspirin (acetylsalicylic acid) associated lncRNAs across 10 cancer types. D, The heatmap for lncRNAs for 10 cancer types. (TIF) [file pcbi.1007540.s006.tif]

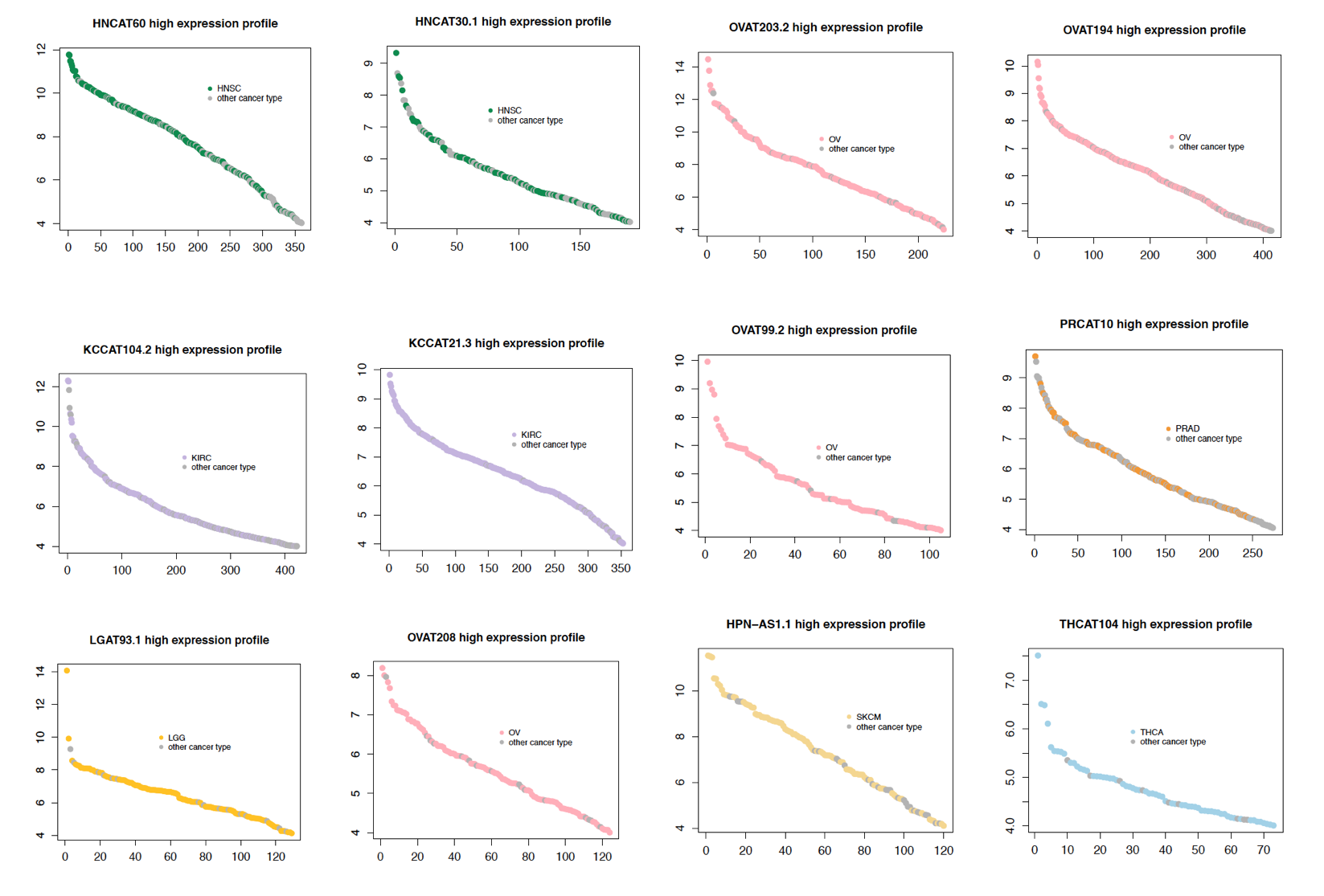

Supplement: S7 Fig — (TIF) [file pcbi.1007540.s007.tif]

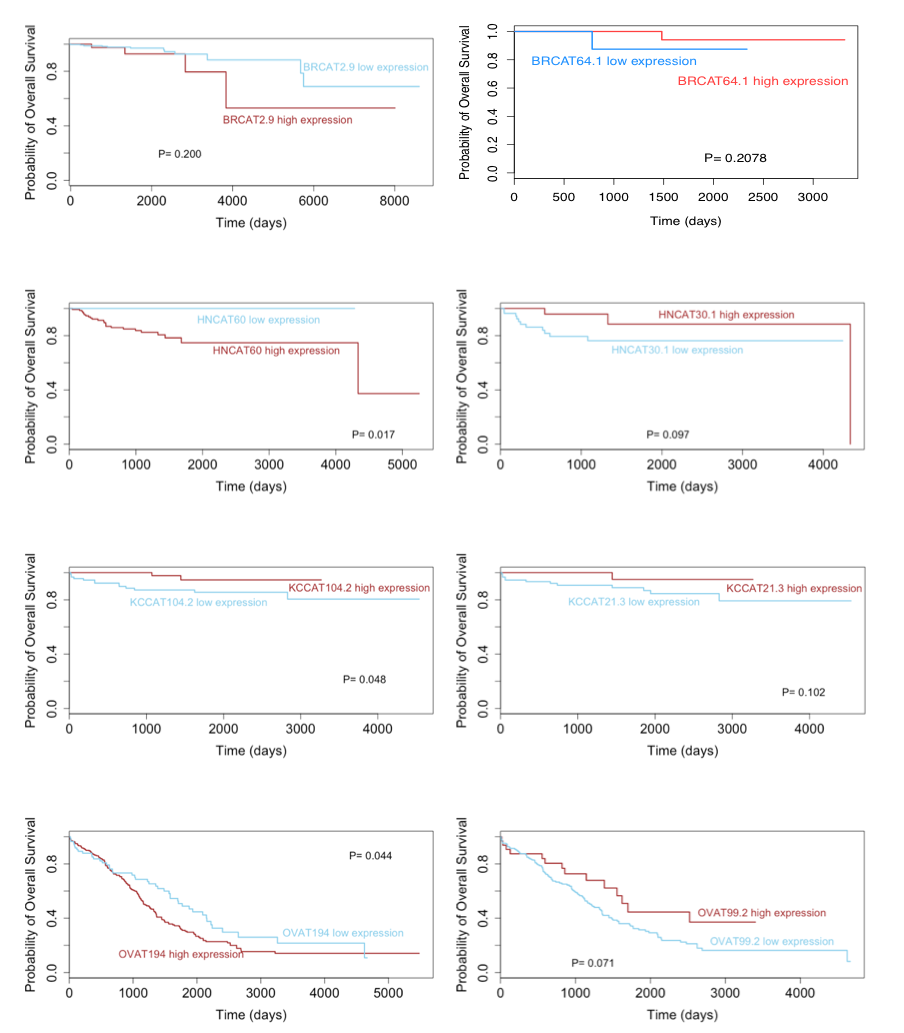

Supplement: S8 Fig — (TIF) [file pcbi.1007540.s008.tif]
