# Supplementary material for: Associating lncRNAs with small molecules via bilevel optimization reveals cancer-related lncRNAs
Source: PLoS Comput Biol. 2019 Dec 26;15(12):e1007540. doi: 10.1371/journal.pcbi.1007540 (PMC6948815; doi:10.1371/journal.pcbi.1007540)
Supplement: S1 Table — (DOCX) [file pcbi.1007540.s009.docx]

Table S1

| Tumor type | No. of matched sample | No. of lncRNA-gene pairs with correlation coefficients larger than 0.7 (0.5) | Percentage of lncRNA-gene pairs with PCC in [0.5-0.7] (0.3-0.5) and predicted correlation score larger than 0.9 |
| --- | --- | --- | --- |
| BRCA | 569 | 2423 | 68.3% |
| HNSC | 332 | 238 (1358) | (49.3%) |
| KIRC | 531 | 200 (933) | (7.2%) |
| LGG | 219 | 3031 | 42.8% |
| LUAD | 400 | 365 (1630) | (47.1%) |
| LUSC | 383 | 323 (1323) | (50.2%) |
| OV | 384 | (39) | (11.6%) |
| PRAD | 217 | 1649 | 63.1% |
| SKCM | 258 | 908 | 60.2% |
| THCA | 521 | 299 (1946) | (56.3%) |
