# Supplementary material for: Associating lncRNAs with small molecules via bilevel optimization reveals cancer-related lncRNAs
Source: PLoS Comput Biol. 2019 Dec 26;15(12):e1007540. doi: 10.1371/journal.pcbi.1007540 (PMC6948815; doi:10.1371/journal.pcbi.1007540)
Supplement: S2 Table — The better results are highlighted in bold. (DOCX) [file pcbi.1007540.s010.docx]

Table S2.

| **lncRNA_symbol** | **gene_symbol** | **cor-eff** | **Predicted scores** |
| --- | --- | --- | --- |
| NEAT1 | ADARB2 | 0.143 | **0.554** |
| HOTAIRM1 | HOXA1 | **0.262** | 0.024 |
| HOTAIRM1 | HOXA4 | 0.631 | **0.996** |
| EMX2OS | EMX2 | **0.782** | 0.417 |
| NEAT1 | ANK2 | 0.127 | **0.531** |
| NEAT1 | CD3D | -0.133 | **0.324** |
| NEAT1 | GFRA3 | -0.367 | **0.444** |
| NEAT1 | MATN2 | 0.123 | **0.500** |
| NEAT1 | MGAT4C | 0.032 | **0.363** |
| NEAT1 | MPPED2 | 0.093 | **0.555** |
| NEAT1 | NDST3 | -0.041 | **0.321** |
| NEAT1 | NME5 | 0.331 | **0.527** |
| NEAT1 | PCDHGC3 | 0.0001 | **0.485** |
| NEAT1 | SLC26A8 | 0.032 | **0.5000** |
| NEAT1 | TCEAL7 | 0.186 | **0.471** |
| NEAT1 | TRIM9 | 0.086 | **0.417** |
| NEAT1 | TSGA10 | 0.315 | **0.533** |
| NEAT1 | ZNF708 | 0.101 | **0.479** |
