# Supplementary material for: Associating lncRNAs with small molecules via bilevel optimization reveals cancer-related lncRNAs
Source: PLoS Comput Biol. 2019 Dec 26;15(12):e1007540. doi: 10.1371/journal.pcbi.1007540 (PMC6948815; doi:10.1371/journal.pcbi.1007540)
Supplement: S3 Table — The literature supports for associations of genes with corresponding type of cancer are suggested. Note: * adjustment p-value less than 0.001. (DOCX) [file pcbi.1007540.s011.docx]

Table S3

| **Drug** | **lncRNA, associated disease, and logFC** | **Overlap gene** | **Shared/enriched GO term and KEGG pathway** |
| --- | --- | --- | --- |
| Estradiol | BRCAT2.9  Breast cancer: 0.661  1.40* | GHITM^1^,  EFTUD1^2^, SQSTM1^3^  PI4K2A, GK  RS: 99.7 | Protein binding/ kinase activity  Metabolic pathway |
| Fulvestrant | CAT354  Breast cancer: 0.838  3.11* | SMARCA5^4^, CDK7^5^, ETV5^6^, TGIF1  RS: 99.4 | transcription factor activity |
| Genistein | CAT1875.2  Breast cancer: 0.01  -0.16 | MT1H^7^, MT1X^7^, SQSTM1^3^, ZDHHC3, COQ10B  RS: 99.7 | protein binding  Mineral absorption |
| LY-294002 | LINC00665.6  Breast cancer: 0.918  3.03* | GFPT1^8^, FDPS^9^, BIK^10^, WAC, PROSC  RS: 99.7 | protein binding  Metabolic pathway |
| LY-294002 | BRCAT64.1  Breast cancer: 0.559  1.40* | GRB10^11^, PPP2R2A^12^, ATXN1^13^, CASK, PDIA4  RS: 99.7 | protein binding |
| Trichostatin A | CAT1425.1  Breast cancer: 0.838  -0.21 | MT1X^7^, HSD17B7^14^ , DHX32, PSD3^15^, ARMCX3  RS: 99.7 | -- |
| Trichostatin A | FAM13A-AS1  Breast cancer: 0.657  -1.72* | BTG1^16^, RPN1, NFKBIA, CCNL1^17^, NEU1  RS: 99.7 | protein binding |
| Alvespimycin | CAT1542.1  Breast cancer: 0.735  0.88 | CLTCL1^18^, FGF5, SRM, FMO4  RS: 99.4 | -- |
| Geldanamycin | BRCAT113  Breast cancer: 0.143  2.25* | SAMSN1, TLR4^19^, FCGR2A^20^, MS4A6A, SLC1A3^21^, C3AR1, TFEC^22^, IL10RA, CD84  RS: 99.7 | protein binding  Infection |
| Tanespimycin | BRCAT59.1  Breast cancer: 0.541  1.25* | PPP1R14B, MBNL2^23^, TIMM8A, RPP40, TCEB3, MRTO4, PFDN2, APRT  RS: 99.8 | protein binding |
| Monorden | BRCAT205  Breast cancer: 0.776  -0.28 | BYSL^24^, CD84, RPP38, SELPLG  RS: 99.4 | protein binding |
| Wortmannin | BRCAT174  Breast cancer: 0.574  0.95 | CD207^25^, MGAT3^26^, ATP4B, NFIX^27^, SCNN1D, LALBA  RS: 99.8 | protein binding  Metabolic pathway |
| Wortmannin | MBNL1-AS1.2  Breast cancer: 0.873  -0.78 | PSMA4, DLG4^28^, BYSL^29^, OR2H1, PNMA2  RS: 99.7 | protein binding |
| Acetylsalicylic acid | CAT1267  Breast cancer: 0.924  1.160* | PHF14, MZF1^30^, PKN2^31^, RSU1^32^  RS: 99.4 | protein binding |
