# Supplementary material for: Associating lncRNAs with small molecules via bilevel optimization reveals cancer-related lncRNAs
Source: PLoS Comput Biol. 2019 Dec 26;15(12):e1007540. doi: 10.1371/journal.pcbi.1007540 (PMC6948815; doi:10.1371/journal.pcbi.1007540)
Supplement: S4 Table — The literature supports for associations of genes with corresponding type of cancer are suggested. Note: * adjustment p-value less than 0.001. (DOCX) [file pcbi.1007540.s012.docx]

Table S4

| **Drug** | **lncRNA, associated disease, and logFC** | **Overlap gene** | **Shared/enriched GO term and KEGG pathway** |
| --- | --- | --- | --- |
| Chlorpromazine | CASK-AS1  HNSC: 0.267  1.47* | IER3, VEGFA, TNFAIP3, HSPA13, STC1  RS: 99.7 | protein binding |
| Fluphenazine | HNCAT60  HNSC: 0.672  5.47* | DHX32, SLC7A11, WHSC1^33^, SC5D, APLP2  RS: 99.8 | protein binding |
| LY-294002 | CAT89.2  HNSC: 0.361  -1.23* | RERE, SCD, SOS2, ADRBK1  RS: 99.4 | -- |
| Trichostatin A | CAT1086  HNSC: 0.821  1.53* | CXCR4, PACSIN2, DCTN4, SOS2  RS: 99.4 | protein binding  Chemokine signaling pathway |
| Thioridazine | CAT904.8  HNSC: 0.491  2.04* | SMARCA5, PACSIN2, JUND, ARFGAP3, SOS2  RS: 99.8 | protein binding  MAPK signaling pathway |
| Valproic acid | EPB41L4A-AS1.1  HNSC: 0.859  3.81* | RERE, RPN1, NFKBIA, COPG1  RS: 99.4 | protein binding |
| Alvespimycin | CAT1203  HNSC: 0.876  0.03 | CLU, PDGFD, NAB1, ZNF76  RS: 99.5 | -- |
| Clozapine | LINC00491.1  HNSC: 0.429  3.72* | CDKL1, ADH5, PSIP1, C4orf27, MOSPD1  RS: 99.9 | -- |
| Geldanamycin | HNCAT274  HNSC: 0.141  0.65 | FCGR2A, RAB33A, C3AR1, TFEC, CD84  RS: 99.8 | protein binding  Staphylococcus aureus infection |
| Monorden | HNCAT30.1  HNSC: 0.211  2.29* | BYSL, NIP7^34^, TIMM17A, METTL1, SELPLG  RS: 99.8 | protein binding |
| Tanespimycin | HNCAT94  HNSC: 0.641  -0.36 | PTPRC, KIAA0226L, LILRA2, GPR65  RS: 99.0 | receptor activity |
| Haloperidol | HNCAT94  HNSC: 0.641  -0.36 | CD37, SIGLEC5, KCNA3, LY75  RS: 99.0 | -- |
| Wortmannin | CAT481  HNSC: 0.773  1.50* | FXYD7, HMX1, ATF5, REG1B, DCC  RS: 99.8 | protein binding |
| Acetylsalicylic acid | HNCAT134  HNSC: 0.532  2.30* | PRSS12, SAMD9, SYNGR3, MEGF8  RS: 99.6 | protein binding |
