# Supplementary material for: Associating lncRNAs with small molecules via bilevel optimization reveals cancer-related lncRNAs
Source: PLoS Comput Biol. 2019 Dec 26;15(12):e1007540. doi: 10.1371/journal.pcbi.1007540 (PMC6948815; doi:10.1371/journal.pcbi.1007540)
Supplement: S5 Table — The literature supports for associations of genes with corresponding type of cancer are suggested. Note: * adjustment p-value less than 0.001. (DOCX) [file pcbi.1007540.s013.docx]

Table S5

| **Drug** | **lncRNA , associated disease, and logFC** | **Overlap gene** | **Shared/enriched GO term and KEGG pathway** |
| --- | --- | --- | --- |
| LY-294002 | KCCAT104.2  KIRC: 0.866  2.82* | ETV5, CNOT8, GRB10^35^, AKAP13, KHNYN  RS: 99.8 | protein binding |
| Trichostatin A | KCCAT374.2  KIRC: 0.521  0.50 | CXCR4, VEGFA, KHNYN, SLC1A4  RS: 99.4 | Cytokine binding  Cytokine-cytokine receptor interaction |
| Alvespimycin | PHKA2-AS1.3  KIRC: 0.185  0.13 | REXO4, ZNF282, SEMA4C, E4F1, TNK2  RS: 99.8 | DNA binding |
| Geldanamycin | KCCAT21.3  KIRC: 0.392  3.80* | FCGR2A, MS4A6A, SLC1A3, IL10RA, CD84  RS: 99.8 | protein binding |
| Tanespimycin | KCCAT21.3  KIRC: 0.392  3.80* | MS4A6A, CD84^35^, FCGR2A, FCGR2C, TRPV2, IL10RA  RS: 99.9 | IgG binding^E^  Tuberculosis^E^ |
| Wortmannin | CAT506  KIRC: 0.888  -0.04 | NPAS3, CAV3, OR2H1, P2RX6, CTRB2, ETV6  RS: 99.9 | -- |
| Sirolimus | CAT1235.2  KIRC: 0.946  0.42 | CLIP2, S100A3, CSNK1D, AATF, ENND3  RS: 99.8 | -- |
| Acetylsalicylic acid | CAT1016  KIRC: 0.596  3.44* | KIR2DL4, STARD5, TYMP, NACAP1, CDKN2A  RS: 99.8 | Bladder cancer |
