# Supplementary material for: Associating lncRNAs with small molecules via bilevel optimization reveals cancer-related lncRNAs
Source: PLoS Comput Biol. 2019 Dec 26;15(12):e1007540. doi: 10.1371/journal.pcbi.1007540 (PMC6948815; doi:10.1371/journal.pcbi.1007540)
Supplement: S6 Table — The literature supports for associations of genes with corresponding type of cancer are suggested. Note: * adjustment p-value less than 0.001. (DOCX) [file pcbi.1007540.s014.docx]

Table S6

| **Drug** | **lncRNA, associated disease and logFC** | **Overlap gene** | **Shared/enriched GO term and KEGG pathway** |
| --- | --- | --- | --- |
| Chlorpromazine | CAT1940  GBM: 0.838  4.00* | ANKRD12, ARFGEF1, SQLE, SATB2  RS: 99.5 | protein binding |
| Fluphenazine | GBAT36  GBM: 0.143  0.33 | CHMP1B, IER3, GPRC5A, TNFAIP  RS: 99.5 | protein binding |
| LY-294002 | CAT33  GBM: 0.846  2.01* | ARFGEF1, SQLE, ANKRD12,  SATB2, RANBP9  RS: 99.9 | protein binding |
| Trichostatin A | CAT1463  GBM: 0.819  -2.53* | SLC1A4, ARFGEF1, H1FX, CANX  RS: 99.5 | -- |
| Thioridazine | CAT1940  GBM: 0.839  4.00* | GOLGB1, ANKRD12, JUND, ARFGEF1  RS: 99.5 | transcription factor activity |
| Valproic acid | LGAT93.1  LGG: 0.621  3.47* | ARFGEF1, CYLD, APLP2, PJA2  RS: 99.5 | protein kinase binding |
| Acetylsalicylic acid | CAT1352.2  LGG: 0.105  -0.01 | DDX60, IFI44L, LAP3, SAMD9, TYMP, FPR2  RS: 99.9 | Metabolic pathways |
| Alvespimycin | LGAT94  LGG: 0.127  2.21* | HERC4, TNF, REXO4, METTL9, ZNF282, PLAC1  RS: 99.9 | DNA binding |
| Clozapine | LGAT76.1  LGG: 0.101  2.42* | ZNF702P, HNRNPM, AATF, COQ10B  RS: 99.4 | -- |
| Geldanamycin | FAM83H-AS1.4  LGG: 0.916  -1.53* | TNFAIP1, WDR76, ELL3, PCBD1  RS: 99.4 | protein binding, |
| Haloperidol | CAT726  Medulloblastoma: 0.667  0.94 | MT1M, HEY1, PSMB5, SERPINB10 | -- |
| Monorden | CAT1783.2  LGG: 0.377  4.31* | VAV3, MGEA5, BST2^36^, ZNF358  RS: 99.4 | -- |
| Tanespimycin | LGAT9  LGG: 0.389  5.41* | SRM, POLR3K, IMP4, CDK4^37^, RPP40  RS: 99.8 | protein binding |
| Wortmannin | HNCAT182.1  HNSC: 0.315  -0.13 | KCNE1, LUZP4, KIR3DX1,  OR2H1, ATP4B  RS: 99.8 | protein binding |
