# Supplementary material for: Associating lncRNAs with small molecules via bilevel optimization reveals cancer-related lncRNAs
Source: PLoS Comput Biol. 2019 Dec 26;15(12):e1007540. doi: 10.1371/journal.pcbi.1007540 (PMC6948815; doi:10.1371/journal.pcbi.1007540)
Supplement: S7 Table — The literature supports for associations of genes with corresponding type of cancer are suggested. Note: * adjustment p-value less than 0.001. (DOCX) [file pcbi.1007540.s015.docx]

Table S7

| **Drug** | **lncRNA , associated disease, and logFC** | **Overlap gene** | **Shared/enriched GO term and KEGG pathway** |
| --- | --- | --- | --- |
| LY-294002 | CAT785  LUAD: 0.833  2.44* | INSIG1, SOS2, BMP2K, ATXN1, ATP2C1  RS: 99.9 | protein binding  Pathways in cancer |
| Trichostatin A | LACAT89  LUAD: 0.857  1.27* | LDLR, VEGFA, HHLA3, KLF6, LRP3  RS: 99.9 | protein binding |
| Trichostatin A | CAT962  LUAD: 0.806  0.86 | SETX, INSIG1, GFPT1, CNL1  RS: 99.7 | protein binding |
| Acetylsalicylic acid | CAT465.2  LUAD: 0.194  0.73 | AKAP5^38^, FZD6, SLC2A4RG, FOXN2, KIAA0355  RS: 99.9 | protein binding |
| Alvespimycin | PART1.4  LUAD: 0.830  -0.75 | CD300A, IRAK3, APBB1IP, TNIP1, ADTRP  RS: 99.9 | protein binding |
| Geldanamycin | CAT194.1  LUAD: 0.662  2.49* | E2F8, TNFAIP1, CHAF1B, PKNOX2  RS: 99.7 | -- |
| Geldanamycin | LACAT27  LUAD: 0.302  0.99 | STX11, DAO, ENC1, L3RA  RS: 99.7 | protein binding |
| Monorden | CAT354  LUAD: 0.838  3.84* | BID, IGF2BP3, BST2, PRKAR1A  RS: 99.7 | protein binding |
| Tanespimycin | LINC00665.6  LUAD: 0.918  0.81 | NME1, PPP1R14B, TMEM147, IMP4, RPP40  RS: 99.9 | protein binding  Ribosome biogenesis in eukaryotes |
| Wortmannin | CAT623.2  LUAD: 0.693  -0.05 | WNT2B, IL1RL1, FUT2, CAV3, SLC22A18AS  RS: 99.9 | -- |
