# Supplementary material for: Associating lncRNAs with small molecules via bilevel optimization reveals cancer-related lncRNAs
Source: PLoS Comput Biol. 2019 Dec 26;15(12):e1007540. doi: 10.1371/journal.pcbi.1007540 (PMC6948815; doi:10.1371/journal.pcbi.1007540)
Supplement: S8 Table — The literature supports for associations of genes with corresponding type of cancer are suggested. Note: * adjustment p-value less than 0.001. (DOCX) [file pcbi.1007540.s016.docx]

Table S8

| **Drug** | **lncRNA, associated disease, and logFC** | **Overlap genes** | **Shared/enriched GO term and KEGG pathway** |
| --- | --- | --- | --- |
| LY-294002 | LSCAT14  LUSC: 0.123  1.71* | DNAJC10, WAC, KCNMB3, GPR137B, SEC62^39^  RS: 99.8 | protein binding  Protein processing in endoplasmic reticulum, |
| Trichostatin A | LSCAT161  LUSC: 0.056  1.31* | DNAJC10, RHOQ, LMAN1, KCNMB3, KLHL24  RS: 99.8 | protein binding  Insulin signaling pathway |
| Acetylsalicylic acid | CAT1406.1  LUSC: 0.545  -2.26* | BAAT, SECISBP2L, TRMT61A, FPR2, FCGR2A  RS: 99.8 | protein binding |
| Alvespimycin | CAT945.1  LUSC: 0.855  -1.03 | NAB1, ID1, FAM49A, MAS1, GSPT1  RS: 99.8 | protein binding |
| Geldanamycin | CAT2180.4  LUSC: 0.882  1.56* | IP6K1, TAOK3, CTDSP2, C3AR1, CD84, FYCO1, PIN4  RS: 99.9 | protein binding |
| Geldanamycin | LSCAT255  LUSC: 0.735  -0.87 | RIN3, C3AR1, CD84, TBC1D2B, FYCO1  RS: 99.8 | protein binding |
| Monorden | CAT257  LUSC: 0.930  -1.64* | CSF3R, CD84, EPB41L3, STX3, FCAR  RS: 99.8 | protein binding |
| Monorden | LINC00511.2  LUSC: 0.725  2.35* | TWISTNB, BID, ENOPH1, HSP90AA1, PNO1  RS: 99.8 | -- |
| Tanespimycin | CAT1149.1  LUSC: 0.521  -1.56* | SRM, MRTO4, MRPL15, IMP4, TIMM8A, RAD1, RUVBL1  RS: 99.9 | protein binding |
| Wortmannin | CAT1744.1  LUSC: 0.405  1.79* | SYNGR4, DRD5, MANF, CD207, MASP1  RS: 99.8 | -- |
