# Supplementary material for: Associating lncRNAs with small molecules via bilevel optimization reveals cancer-related lncRNAs
Source: PLoS Comput Biol. 2019 Dec 26;15(12):e1007540. doi: 10.1371/journal.pcbi.1007540 (PMC6948815; doi:10.1371/journal.pcbi.1007540)
Supplement: S9 Table — The literature supports for associations of genes with corresponding type of cancer are suggested. Note: * adjustment p-value less than 0.001. (DOCX) [file pcbi.1007540.s017.docx]

Table S9.

| **Drug** | **lncRNA , associated disease, and logFC** | **Overlap genes** | **Shared/enriched GO term and KEGG pathway** |
| --- | --- | --- | --- |
| LY-294002 | OVAT208  OV: 0.161  1.53* | INSIG1, SCD, GDF15^40^, GPR137B, HHLA3  RS: 99.7 | protein binding |
| Trichostatin A | CAT404  OV: 0.141  -2.44* | HBP1, MBNL2, MAP1LC3B, SOS2, MGAT2, PJA2  RS: 99.8 | protein binding  Metabolic pathways |
| Acetylsalicylic acid | CAT2047  OV: 0.705  1.43* | AP2S1, TMEM143, OX10, PKN2, RPS8, MEN1  RS: 99.8 | -- |
| Alvespimycin | OVAT194  OV: 0.855  3.74* | IGF2BP3^41^, CRYZ, TRPC4, ACP2, AVEN  RS: 99.7 | protein binding |
| Geldanamycin | CAT403.2  OV: 0.663  3.01* | CRYZ, ANKRD10, CCDC86, FGF5, ULK1  RS: 99.7 | protein binding |
| Monorden | CAT2197  OV: 0.768  5.00* | OXTR, TIMM17A, RBM5, MGEA5, CHD3  RS: 99.7 | -- |
| Monorden | OVAT166.1  OV: 0.651  4.93* | CRYZ, TIMM17A, MGEA5, CHD3, VILL  RS: 99.7 | -- |
| Tanespimycin | OVAT203.2  OV: 0.915  4.17* | CASP1, POLR3K, RAD1, PNOC^42^, VAV3, TFEC, PLSCR1  RS: 99.9 | -- |
| Wortmannin | OVAT99.2  OV: 0.232  1.38* | PRKCQ^43^, ITIH3, KCNMB2, PF4, KRT3  RS: 99.7 | Insulin signal pathway |
