# Supplementary material for: Associating lncRNAs with small molecules via bilevel optimization reveals cancer-related lncRNAs
Source: PLoS Comput Biol. 2019 Dec 26;15(12):e1007540. doi: 10.1371/journal.pcbi.1007540 (PMC6948815; doi:10.1371/journal.pcbi.1007540)
Supplement: S10 Table — The literature supports for associations of genes with corresponding type of cancer are suggested. Note: * adjustment p-value less than 0.001. (DOCX) [file pcbi.1007540.s018.docx]

Table S10.

| **Drug** | **lncRNA , associated disease, and logFC** | **Overlap genes** | **Shared/enriched GO term and KEGG pathway** |
| --- | --- | --- | --- |
| LY-294002 | PCA3.4  PRAD: 0.973  LncRNADisease  10.6* | WIPI1^44^, EIF2AK3, HSPA13, HHLA3, SEC24D  RS: 99.7 | protein binding  Protein processing in endoplasmic reticulum |
| LY-294002 | LINC00958.9  (PRAD: 0.913)  -0.12 | GFPT1, EIF2AK3, SPA13, SEC24D, UGCG  RS: 99.7 | -- |
| Trichostatin A | PRCAT77  PRAD: 0.767  1.22* | PPP2R2D, SETX, STAG2, SMARCA5, HHLA3  RS: 99.7 | protein binding |
| Acetylsalicylic acid | PRCAT10  PRAD: 0.590  2.97* | MZF1^45^, NACAP1, MORN1, TRMT61A, SLC2A4RG  RS: 99.7 | transcription factor activity |
| Alvespimycin | PRCAT32.3  PRAD: 0.658  3.94* | CD300A, TNF, FAM49A, PDZD2 | protein binding |
| Geldanamycin | CAT1212  PRAD: 0.878  3.58* | MSH6, NDUFS6, STX11, IL10RA, CFLAR  RS: 99.7 | protein binding |
| Monorden | BRCAT1.5  breast: 0.899  -0.16 | IL5RA, PLOD1, SULT1A1, MGEA5, METTL1, NUDT11  RS: 99.9 | -- |
| Tanespimycin | CAT1337.2  PRAD: 0.884  1.21* | METTL13, PNP, E2F8, PRMT2, PLSCR1  RS: 99.7 | -- |
| Tanespimycin | PRCAT268  PRAD: 0.761  0.70 | MT2A, PNP, MGEA5, MT1F, MT1E  RS: 99.7 | zinc ion binding  Mineral absorption |
| Wortmannin | PRCAT275  PRAD: 0.124  -0.22 | SNAI1, IFNA4, NOL9, IL1B, DACT1  RS: 99.7 | protein binding  Toll-like receptor signaling pathway |
