# Supplementary material for: Associating lncRNAs with small molecules via bilevel optimization reveals cancer-related lncRNAs
Source: PLoS Comput Biol. 2019 Dec 26;15(12):e1007540. doi: 10.1371/journal.pcbi.1007540 (PMC6948815; doi:10.1371/journal.pcbi.1007540)
Supplement: S11 Table — The literature supports for associations of genes with corresponding type of cancer are suggested. Note: * adjustment p-value less than 0.001. (DOCX) [file pcbi.1007540.s019.docx]

Table S11.

| **Drug** | **lncRNA , associated disease, and logFC** | **Overlap genes** | **Shared/enriched GO term and KEGG pathway** |
| --- | --- | --- | --- |
| LY-294002 | JPX.1  SKCM: 0.811  -1.91* | PJA2, SERINC1, NBR1, ATXN1  RS: 99.4 | protein binding, |
| Tretinoin | LINC00518.7  SKCM: 0.899  7.57* | CHMP1B, GFPT1, SEC24D, EIF4G3  RS: 99.4 | -- |
| Trichostatin A | CAT51.3  SKCM: 1.000  -1.69* | ARIH1, NFIL3, SOS2, H1FX, KCNMB3  RS: 99.7 | DNA binding  Insulin signaling pathway |
| Acetylsalicylic acid | MEAT119  SKCM: 0.734  -2.10* | SECISBP2L, DDR2, LRCH1, TAOK1, KIF3A  RS: 99.7 | ATP binding |
| Alvespimycin | CAT1388.2  SKCM: 0.571  -0.64 | CD300A, APBB1IP, CRYZ, KLK12, RBM4B  RS: 99.7 | -- |
| Geldanamycin | CAT2215.1  SKCM: 0.861  -2.58* | CRYZ, TLR4, FCGR2A, MS4A6A, IP6K1, TNFAIP1, C3AR1, TFEC  RS: 99.7 | protein binding |
| Geldanamycin | HPN-AS1.1  SKCM: 0.652  3.04* | E2F8^47^, KRT19, TNFAIP1, IGFBP1, ZDHHC13  RS: 99.7 | protein binding |
| Monorden | CAT1729.4  SKCM: 0.774  -2.52* | TFEC, CD38, CD84, DNAJA4, SELPLG  RS: 99.7 | protein binding |
| Tanespimycin | CAT1729.4  SKCM: 0.774  -2.52* | CD84, LILRA2, TFEC, SLA, DNAJA4  RS: 99.7 | protein binding |
| Wortmannin | CAT1118  SKCM: 0.268  -0.89 | FUT2, PSG3, GAPDHS, TP53TG5, ATP4B  RS: 99.7 | Metabolic pathways |
