# Supplementary material for: Associating lncRNAs with small molecules via bilevel optimization reveals cancer-related lncRNAs
Source: PLoS Comput Biol. 2019 Dec 26;15(12):e1007540. doi: 10.1371/journal.pcbi.1007540 (PMC6948815; doi:10.1371/journal.pcbi.1007540)
Supplement: S12 Table — The literature supports for associations of genes with corresponding type of cancer are suggested. Note: * adjustment p-value less than 0.001. (DOCX) [file pcbi.1007540.s020.docx]

Table S12.

| **Drug** | **lncRNA , associated disease, and logFC** | **Overlap genes** | **Shared/enriched GO term and KEGG pathway** |
| --- | --- | --- | --- |
| LY-294002 | THCAT135  THCA: 0.088  1.97* | SETX, GOLGB1, ANKRD12, SEC62, RHOT2  RS: 99.8 | -- |
| Trichostatin A | THCAT104  THCA: 0.031  1.47* | SEC24D, KIF3C, BMP2K, CYLD, JAK2^46^, TES  RS: 99.9 | protein binding |
| Acetylsalicylic acid | CAT1773  THCA: 1.000  -0.41 | IFI44L, NACAP1, PALMD, DDR2, SOX10, WWTR1  RS: 99.9 | protein binding |
| Alvespimycin | PTCSC3.1  THCA: 0.651  LncRNADisease  5.28* | ZNF79, REXO2, MAVS, PSORS1C1  RS: 99.5 | nucleic acid binding |
| Alvespimycin | LRP4-AS1.2  THCA: 0.226  1.32* | OXTR, PIAS3, EDN1, SEMA4C, SORS1C1, TNK2  RS: 99.9 | protein binding |
| Geldanamycin | CAT12.1  THCA: 0.721  2.88* | HRH1, OXTR, GPD1L, CCNA1, CDH11  RS: 99.8 | -- |
| Monorden | CAT179  THCA: 0.903  1.79* | ELMO1, HSP90AA1, BLVRA, SYNGR2, FAM69A  RS: 99.8 | protein binding |
| Tanespimycin | CAT1458.1  THCA: 0.189  -0.11 | CASP1, PNP, CGR2A, TRPV2, TFEC, IL10RA  RS: 99.9 | -- |
| Wortmannin | LINC00958.9  THCA: 0.913  5.86* | CAMKK2, CKMT2, MAK16, ELANE, KCNQ2, GCM1  RS: 99.9 | -- |
