# Supplementary material for: Associating lncRNAs with small molecules via bilevel optimization reveals cancer-related lncRNAs
Source: PLoS Comput Biol. 2019 Dec 26;15(12):e1007540. doi: 10.1371/journal.pcbi.1007540 (PMC6948815; doi:10.1371/journal.pcbi.1007540)
Supplement: S13 Table — (DOCX) [file pcbi.1007540.s021.docx]

Table S13

| Drug/  reported cancer | **lncRNA** | **Target Gene** | **Cancer Type** |
| --- | --- | --- | --- |
| Estradiol  Breast^48^ | BRCAT2.9* | EFTUD1^2^ | BRCA |
| LY-294002  Breast^49^ | BRCAT64.1* | PPP2R2A^12^ | BRCA |
| Fluphenazine  Myeloma^50^ | HNCAT60* | WHSC1^33^ | HNSC |
| Monorden | HNCAT30.1* | NIP7^34^ | HNSC |
| LY-294002  Renal^51^ | KCCAT104.2* | GRB10^35^ | KIRC |
| Tanespimycin  Renal^52^ | KCCAT21.3* | CD84^35^ | KIRC |
| Valproic acid  Brain^53^ | LGAT93.1* | PJA2 | LGG |
| LY-294002  Ovarian^55^ | OVAT208* | GDF15^40^ | OV |
| Tanespimycin  Ovarian^56^ | OVAT203.2* | PNOC^41^ | OV |
| Alvespimycin  Ovarian^57^ | OVAT194* | IGF2BP341 | OV |
| Wortmannin  Ovarian^58^ | OVAT99.2* | PRKCQ^43^ | OV |
| LY-294002  Prostate^59^ | PCA3.4* | WIPI1^44^ | PRAD |
| Geldanamycin^60^ | HPN-AS1.1 | E2F8^47^ | SKCM |
| Trichostatin A  Thyroid^61^ | THCAT104* | JAK2^46^ | THCA |
