# Supplementary material for: Associating lncRNAs with small molecules via bilevel optimization reveals cancer-related lncRNAs
Source: PLoS Comput Biol. 2019 Dec 26;15(12):e1007540. doi: 10.1371/journal.pcbi.1007540 (PMC6948815; doi:10.1371/journal.pcbi.1007540)
Supplement: S14 Table — Note: * adjusted p-value less than 0.001; # survival p-value less than 0.25; ## survival p-value less than 0.05. (DOCX) [file pcbi.1007540.s022.docx]

Table S14

| Drug/  reported cancer | lncRNA | Target Gene | Biological pathway | Cancer Type |
| --- | --- | --- | --- | --- |
| Estradiol  breast | BRCAT2.9^*#^ | EFTUD1^2^ | Ribosome biogenesis | BRCA |
| LY-294002  Breast | BRCAT64.1*^#^ | PPP2R2A^12^ | Adrenergic signaling in cardiomyocytes | BRCA |
| Fluphenazine  myeloma | HNCAT60^*##^ | WHSC1^45^ | Transcriptional misregulation in cancer | HNSC |
| Monorden  -- | HNCAT30.1^*#^ | NIP7^46^ | Ribosome biogenesis | HNSC |
| LY-294002  breast/prostate/ ovarian/renal/lung^54^ | KCCAT104.2^*##^ | GRB10^47^ | Insulin receptor signaling pathway | KIRC |
| Tanespimycin  leukemia^62^/renal/ovary | KCCAT21.3^*#^ | CD84^47^ | Immune response | KIRC |
| Valproic acid  cervical^63^/melanoma^64^/ breast^65^/brain | LGAT93.1^*##^ | PJA2 | Essential for PKA-mediated long-term memory processes | LGG |
| Alvespimycin  breast^57^/ovarian | OVAT194^*##^ | IGF2BP3^53^ | Regulation of cytokine biosynthetic process | OV |
| Wortmannin  breast^66^/ovarian/pancreas^67^ | OVAT99.2^*#^ | PRKCQ^55^ | NF-kappa B signaling pathway  Inflammatory mediator regulation of TRP channels | OV |
