# Supplementary material for: Associating lncRNAs with small molecules via bilevel optimization reveals cancer-related lncRNAs
Source: PLoS Comput Biol. 2019 Dec 26;15(12):e1007540. doi: 10.1371/journal.pcbi.1007540 (PMC6948815; doi:10.1371/journal.pcbi.1007540)
Supplement: S15 Table — (DOCX) [file pcbi.1007540.s023.docx]

Table S15

| drug | BRCA | HNSC | KIRC | LGG | LUAD | LUSC | OV | PRAD | SKCM | THCA |
| --- | --- | --- | --- | --- | --- | --- | --- | --- | --- | --- |
| Doxorubicin | CAT2271  Cervical  0.877 | CAT829  HNSC  0.226 | CAT1510  KICH  0.965 | CAT2185.2  LGG  0.193 | CAT871  LUAD  0.336 | CAT244  LUSC  1.000 | CAT542.1  OV  0.813 | CAT1921.2  PRAD  0.309 | MEAT20.2  SKCM  0.636 | THCAT219.3  THCA  0.016 |
| Paclitaxel | CAT2278  Breast  0.749 | BRCAT177  Breast  0.462 | CAT1510  KICH  0.965 | CAT1499.2  Medulloblastoma  0.552 | CAT1212  LUAD  0.878 | CAT699.2  LUSC  0.492 | OVAT250  OV  0.023 | CAT1947.1  PRAD  0.843 | CAT565  SKCM  0.671 | CAT465.1  THCA  0.546 |
| Carboplatin | CAT2164.2  Breast  0.979 | CAT228.2  KIRC  0.847 | CAT1089.3  KIRC  0.808 | HICLINC349.4  NA | LINC00152.3  LUAD  0.366 | CAT85  LUSC  0.126 | CAT551  BRCA  0.642 | PRCAT222  PRAD  0.249 | MEAT97  SKCM  0.770 | CAT1532  THCA  0.946 |
| Gemcitabine | BRCAT1.2  Breast  0.932 | CAT1138  HNSC  0.138 | KCCAT40  KIRC  0.974 | LINC00320.3  LGG  0.184 | LACAT238  LUAD  0.336 | CAT111.2  LUAD  0.496 | WT1-AS.1  OV  0.349 | PRCAT171  PRAD  0.182 | MEAT70  SKCM  0.602 | THCAT550  THCA  0.480 |
| Docetaxel | CAT1235.1  Breast  0.970 | HNCAT286  HNSC  0.052 | CAT9.2  KIRC  0.526 | LGAT107  LGG  0.532 | CAT969.2  LUAD  0.260 | CAT400.1  OV  0.779 | CAT586.2  BRCA  0.735 | FAM222A-AS1.2  PRAD  0.105 | OVAT20.5  OV  0.360 | CAT373.1  THCA  0.086 |
| Fluorouracil | CAT967  Breast  0.734 | HNCAT232  HNSC  0.054 | KPCAT90.1  KIRP  0.753 | CAT1168.2  LGG  0.340 | LGAT64  LGG  0.234 | CAT1882  LUSC  0.552 | CAT1433.1  OV  0.871 | ATP13A4-AS1  HNSC  0.808 | CAT1177.1  SKCM  0.185 | THCAT417  THCA  0.019 |
| Leucovorin | CAT987  KIRC  0.813 | CAT387  HNSC  0.519 | KCCAT40  KIRC  0.974 | CAT1371.2  LGG  0.284 | CAT608  LUAD  0.471 | LACAT185.1  LUAD  0.669 | CAT1534.2  BRCA  0.936 | CAT2253.2  PRAD  0.672 | MEAT48.1  SKCM  0.675 | OSTM1-AS1.4  KIRC  0.475 |
| Temozolomide | CAT366.3  Breast  0.912 | CAT1840.1  HNSC  0.732 | KCCAT40  KIRC  0.974 | HICLINC353.1  NA | LSCAT95  LUSC  0.298 | CAT2009.2  LUSC  0.503 | CAT885.2  OV  0.162 | CAT1921.2  PRAD  0.309 | CAT2008  SKCM  0.435 | THCAT284.2  THCA  0.193 |
| Pemetrexed | BRCAT157  Breast  0.063 | CAT194.2  HNSC  0.855 | CAT1638  KIRC  0.583 | CAT821.2  LGG  0.955 | CLDN10-AS1.3  LUAD  0.134 | KCCAT128.2  KIRC  0.688 | CAT983.1  OV  0.590 | CAT2268  PRAD  0.943 | CAT421.3  SKCM  0.294 | OSTM1-AS1.4  KIRC  0.475 |
| Dacarbazine | CAT36  Breast  0.961 | CAT464  HNSC  0.546 | KCCAT673.1  KIRC  0.544 | MIR4435-1HG.3  LGG  0.910 | CAT969.2  LUAD  0.260 | CAT631  LUSC  0.758 | OVAT210  OV  0.411 | CAT2268  PRAD  0.943 | MEAT46  SKCM  0.794 | THCAT583  THCA  0.450 |
